# Comparing the Catalytic and Structural Characteristics of a ‘Short’ Unspecific Peroxygenase (UPO) Expressed in *Pichia pastoris* and *Escherichia coli*

**DOI:** 10.1002/cbic.202200558

**Published:** 2022-11-30

**Authors:** Wendy X. Q. Robinson, Tamara Mielke, Benjamin Melling, Anibal Cuetos, Alison Parkin, William P. Unsworth, Jared Cartwright, Gideon Grogan

**Affiliations:** ^1^ York Structural Biology Laboratory Department of Chemistry University of York Heslington York YO10 5DD UK; ^2^ Department of Biology University of York Heslington York YO10 5DD UK

**Keywords:** biocatalysis, hydroxylation, oxygenases, *Pichia pastoris*, unspecific peroxygenases

## Abstract

Unspecific peroxygenases (UPOs) have emerged as valuable tools for the oxygenation of non‐activated carbon atoms, as they exhibit high turnovers, good stability and depend only on hydrogen peroxide as the external oxidant for activity. However, the isolation of UPOs from their natural fungal sources remains a barrier to wider application. We have cloned the gene encoding an ‘artificial’ peroxygenase (artUPO), close in sequence to the ‘short’ UPO from *Marasmius rotula* (*Mro*UPO), and expressed it in both the yeast *Pichia pastoris* and *E. coli* to compare the catalytic and structural characteristics of the enzymes produced in each system. Catalytic efficiency for the UPO substrate 5‐nitro‐1,3‐benzodioxole (NBD) was largely the same for both enzymes, and the structures also revealed few differences apart from the expected glycosylation of the yeast enzyme. However, the glycosylated enzyme displayed greater stability, as determined by nano differential scanning fluorimetry (nano‐DSF) measurements. Interestingly, while artUPO hydroxylated ethylbenzene derivatives to give the (*R*)‐alcohols, also given by a variant of the ‘long’ UPO from *Agrocybe aegerita* (*Aae*UPO), it gave the opposite (*S*)‐series of sulfoxide products from a range of sulfide substrates, broadening the scope for application of the enzymes. The structures of artUPO reveal substantial differences to that of *Aae*UPO, and provide a platform for investigating the distinctive activity of this and related'short’ UPOs.

## Graphical abstract

**Unspecific peroxygenases** (UPOs) are valuable tools for the oxygenation of non‐activated carbon atoms, as they exhibit high turnovers, good stability and depend only on hydrogen peroxide as the external oxidant for activity. We have cloned the gene encoding an ‘artificial’ peroxygenase (artUPO), close in sequence to the ‘short’ UPO from *Marasmius rotula* (*Mro*UPO), and expressed it in both the yeast *Pichia pastoris* and *E. coli* to compare the catalytic and structural characteristics of the enzymes produced in each system.

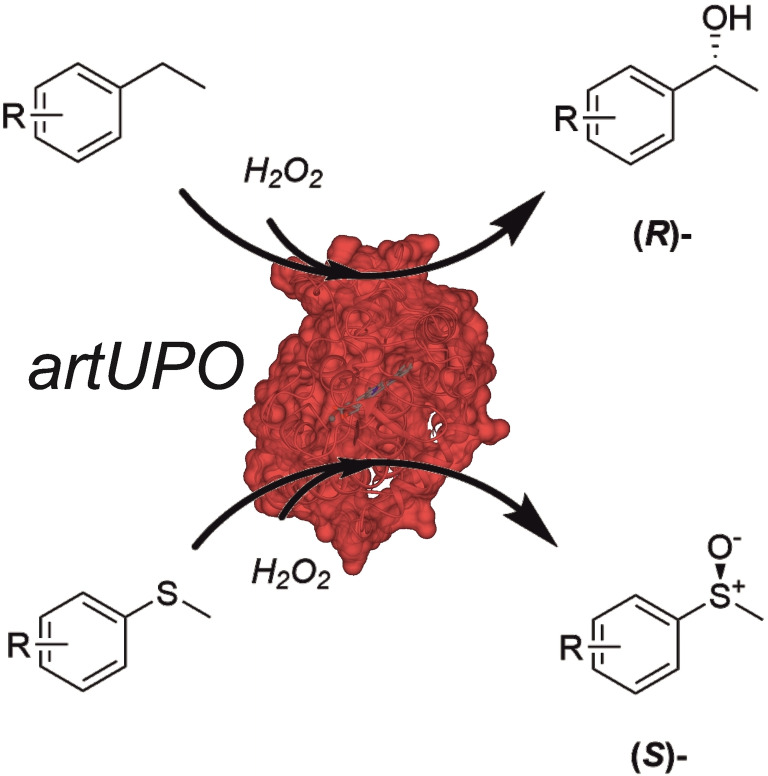

## Introduction

**Scheme 1:**
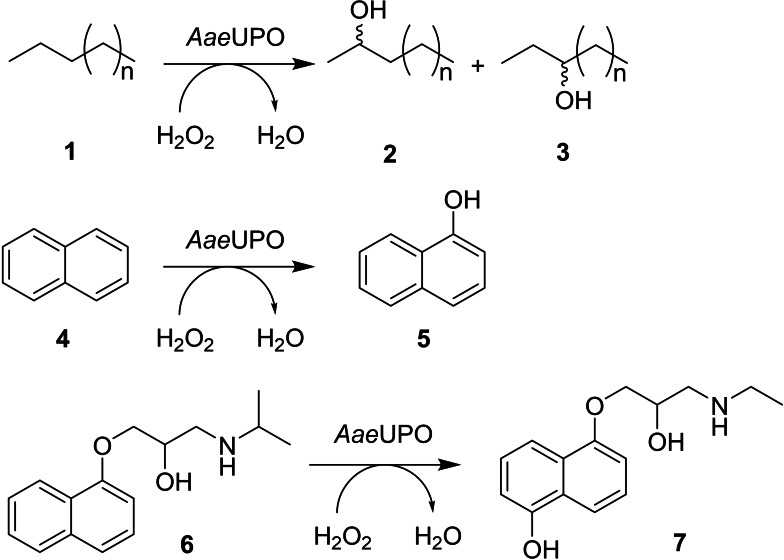
Some oxygenations of substrates catalysed by *Aae*UPO.

Biocatalysis offers many advantages in respect of the oxygenation of organic molecules, as the transformations catalysed often exhibit high degrees of regio‐ and stereoselectivity, and can be achieved under conditions consonant with the principles of sustainable chemistry.[^1^, ^2^, ^3^] Biocatalytic oxygenations can be accomplished by a range of enzymes, exhibiting a host of mechanisms, including those that are dependent on 2‐oxoglutarate,^[4]^ flavins^[5]^ or heme. In the last case, the enzymes that have been most studied for their applications in biocatalysis are the cytochromes P450 (P450s),[^6^, ^7^, ^8^, ^9^] a large family of heme‐thiolate enzymes that selectively oxygenate a range of simple to complex organic molecules from alkanes^[10]^ and alkenes^[11]^ and aromatic nuclei^[12]^ through to terpenes,^[13]^ steroids^[14]^ and pharmaceuticals.^[15]^ Despite the range of enzymes and specificities available, P450s also possess disadvantages with respect to their application, as they are dependent upon an expensive nicotinamide cofactor (NAD(P)/H) for activity, as well as one or more electron transfer proteins to channel electrons from the cofactor to the heme for the oxygenation mechanism to occur. While the application of fused P450 systems, such as P450BM3 from *Bacillus megaterium*,^[16]^ can help to meet these challenges, P450s can still suffer from poor turnover rates and stability. In the context of preparative biotransformations therefore, it is of interest to identify complementary systems for oxygenations that do not suffer from the disadvantages of P450 catalysis. Unspecific peroxygenases (UPOs)[^17^, ^18^] are a class of heme‐thiolate oxygenases from fungi that have similar catalytic scope to P450s, but are dependent only upon hydrogen peroxide (H_2_O_2_) as the external oxidant. Since the identification and characterisation of the first enzyme from *Agrocybe aegerita* (*Aae*UPO) by Hofrichter and co‐workers,[^19^, ^20^, ^21^] this UPO, expressed either from the fungus or in yeasts such as *Saccharomyces cerevisiae* and *P. pastoris*,[^22^, ^23^] has been shown to display a wide spectrum of substrate oxygenations, from aliphatic hydrocarbons **1**,^[24]^ to aromatics such as naphthalene **4** ^[25]^ and drug compounds, including propanolol **6** ^[26]^ (Scheme 1) reminiscent of the range of reactivity displayed by their P450 counterparts.

A recent survey by Faiza and co‐workers^[27]^ explored the diversity of UPOs within sequence databases and was able to identify two major groups of enzymes: ‘long’ UPOs (Group I), typified by *Aae*UPO, of approximately 45 kDa molecular weight, and ‘short’ UPOs (Group II), typified by the enzyme from *Marasmius rotula* (*Mro*UPO), which are of approximately 29 kDa. Recent years have seen the discovery and application of various long[^28^, ^29^, ^30^, ^31^] and short[^32^, ^33^] UPOs and illustrated the diversity of activity that may be accessed by using different enzymes. Despite their enormous potential, access to UPOs is, however, limited by a number of factors. Occurring naturally in eukaryotes, the enzymes are glycosylated, as revealed by the structures of *Aae*UPO[^34^, ^35^] and *Mro*UPO (PDB 5FUJ, 5FUK). While the necessity of the glycosylation for folding or activity of the enzyme has yet to be fully established, there are no reports of the functional expression of long UPOs such as *Aae*UPO in the more convenient *E. coli* expression systems. However, short UPOs, such as *Mro*UPO, have been successfully expressed in functional form in *E. coli*,^[36]^ opening up the possibility of both more accessible expression and also easier directed evolution experiments. However, a direct comparison of the catalytic characteristics of any UPO expressed in yeast and bacteria, in order to evaluate the possible differences in activity or properties, has not yet been presented.

In this study, we have cloned the gene encoding an ‘artificial’ short UPO (artUPO)^[37]^ close in sequence to *Mro*UPO, and expressed it in both *Pichia pastoris* and *E. coli*. This enzyme, when produced recombinantly in *Aspergillus*, was reported to have greater activity and stability than native *Mro*UPO.^[37]^ We have purified the enzyme from both sources and compared both their catalytic characteristics and structures using a mixture of kinetics studies, biotransformations and X‐ray crystallography. We have determined that, while kinetic and structural characteristics appear to be largely conserved in the enzyme produced in bacteria, regardless of the absence of glycosylation, it appears significantly less stable than the enzyme produced from yeast in biotransformation reactions. Either enzyme was, however, active in the transformation of a range of standard UPO substrates to optically enriched products.

## Results and Discussion

### Cloning and expression of artUPO in P. pastoris (artUPO_yeast_)

The artUPO sequence (Figure S1) was reported^[37]^ as having been synthesised based on the *Mro*UPO and other peroxygenase sequences, and shares 73 % sequence identity with that of the *Mro*UPO deposited in the PDB (5FUJ, 5FUK) (Figure S1). The gene encoding this artUPO (Figure S2) was synthesised and ligated into a pPICZα construct that we had previously assembled for the expression of *Aae*UPO.^[38]^ That construct featured a nine‐point mutant of native *Aae*UPO (PaDa‐I) that had been shown by Alcalde and co‐workers to display both superior expression and activity compared to the wild type.[^22^, ^23^] Four mutations within the signal sequence had been shown to be essential for expression in *Pichia pastoris*, as the wild‐type gene was not expressed well in the yeast. In order to construct the vector for artUPO expression in this study, the *Aae*UPO coding sequence was excised and the sequence of the artUPO ligated into the plasmid downstream of the 4‐point mutant signal sequence of *Aae*UPO‐PaDa‐I. The X‐33 strain of *Pichia pastoris* was transformed with this plasmid and western blot analysis of expression trials indicated that ‘artUPO_yeast_’ was expressed by the strain (Figure S3, with a maximum accumulation of enzyme after 24–32 h fermentation, after which the amount of signal from the supernatant declined. The expression strategy resonates with that recently applied by Weissenborn and co‐workers,[^30^, ^31^] in which the signal peptides of some UPOs were found to enable the expression of non‐cognate UPO sequences in experiments with genetic chimeras. Following a 32 h incubation of a 200 mL fermentation, the cells were removed by centrifugation, and the supernatant recovered for assay and further purification. The yield of enzyme corresponded to approximately 1700 U L^−1^ [as assayed with the peroxidase substrate 2,2′‐azino‐bis(3‐ethylbenzothiazoline‐6‐sulfonic acid (ABTS)] of fermentation medium, which is significantly lower than, for example the 232,000 U L^−1^ cited for *Aae*UPO‐PaDa‐I by Molina‐Espeja and co‐workers.^[23]^ This may reflect both of the use of the non‐cognate, non‐optimised signal peptide in expression, but also the much lower specific activity of artUPO for ABTS compared to *Aae*UPO and its variant (*vide infra*). Although the coding sequence used contained a histidine tag, the expressed artUPO_yeast_ did not bind to an NiNTA column. Hence, the supernatant was applied to a MonoQ column, resulting in significant enrichment and purification (Figure S4A). artUPO_yeast_ was then further purified using gel filtration (Figure S4B).

### Cloning and expression of artUPO in E. coli (artUPO_bact_)

The artUPO gene, minus the signal sequence, was also subjected to codon optimisation for expression in *E. coli* and the gene ordered ligated into the pET‐28a(+) vector (Figure S5). In this case, the gene was successfully expressed in the soluble fraction of *E. coli* Rosetta pLysS (DE3) cells following induction with IPTG, 5‐aminolevulinic acid (5ALA) and iron chloride in the mode of successful P450 expression used previously in our group.^[39]^ ‘artUPO_bact_’ was purified in a more straightforward manner than artUPO_yeast_ using NiNTA chromatography followed by size exclusion, to give the pure protein (Figure S6). The yield as measured after NiNTA chromatography was 6.6 mg L^−1^ cell growth, which is comparable to the figure of 10 mg L^−1^ of pure protein cited by Martínez and co‐workers for *Mro*UPO expressed in *E. coli*.^[40]^

### UV spectroscopy and kinetics

**Figure 1:**
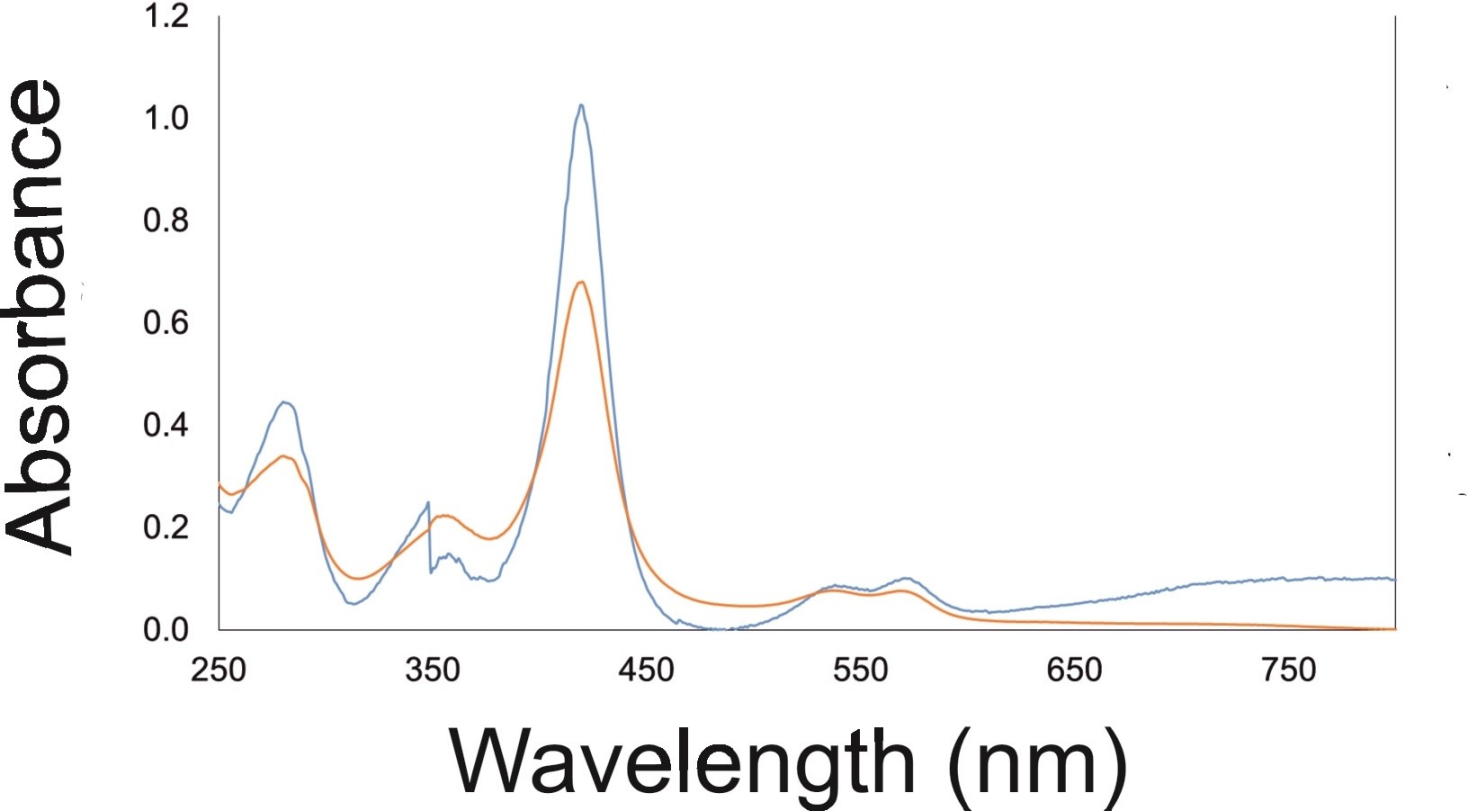
UV/visible spectra of artUPO_yeast_ (blue line) and artUPO_bact_ (orange line) showing Soret peak at 418 nm and charge transfer bands.

Pure artUPO_yeast_ and artUPO_bact_ were analysed using UV spectrophotometry (Figure 1) and displayed spectroscopic characteristics, including a peak at 418 nm, consistent with heme iron ligation to the thiolate ligand of a cysteine residue. artUPO_yeast_ and artUPO_bact_ displayed R_Z_ (Reinheitszhl) values (A_418_/A_280_) of approximately 3 and 2, respectively, possibly indicative of greater heme occupancy within the artUPO_yeast_ preparation.

Each enzyme was subjected to spectrophotometric assays in order to determine kinetic constants for both peroxidase (one electron transfer) and peroxygenase (two electron transfer) activity using ABTS or 5‐nitro‐1,3‐benzodioxole NBD as substrates (Figure S7A−S7D). The kinetic constants were compared with values obtained for wt‐*Aae*UPO,^[22]^ a native *Mro*UPO that had been purified from the *Marasmius* fungus^[32]^ and the short *Hsp*UPO from *Hypoxylon* sp. EC38 (Table 1).^[33]^

**Table 1:** Kinetic constants for wt‐*Aae*UPO, native *Mro*UPO, artUPO_yeast_ and artUPO_bact_ using ABTS or NBD as peroxidase and peroxygenase substrates, respectively.

	ABTS			NBD		
Enzyme	*k*_cat_ [s^−1^]	*K*_M_ [μM]	*k*_cat_/*K*_M_ [μM^−1^ s^−1^]	*k*_cat_ [s^−1^]	*K*_M_ [μM]	*k*_cat_/*K*_M_ [μM^−1^ s^−1^]
wt‐*Aae*UPO^[22]^	221	25	8.84	219	684	0.32
Native *Mro*UPO^[32]^	25	71	0.35	n.r.	n.r.	n.r.
*Hsp*UPO^[33]^	17	30	0.57	10	18	0.57
artUPO_yeast_	48	52	0.92	11	226	0.05
artUPO_bact_	45	35	1.29	5	106	0.05

n.r.=not reported.

The short enzymes artUPO_yeast_, artUPO_bact_ and indeed *Hsp*UPO^[33]^ displayed lower *k*_cat_ values than wt‐*Aae*UPO for both peroxidase and peroxygenase reactions. artUPO_bact_ was a little more active than artUPO_yeast_ and also native *Mro*UPO for peroxidase activity towards ABTS, as a result of a lower measured *K*_M_. While the reason for the difference in peroxidase activity between artUPO_bact_ and artUPO_yeast_ is not clear, a recent molecular dynamics study of non‐glycosylated and glycosylated forms of the heme‐containing horseradish peroxidase by Bertoša and co‐workers^[41]^ suggests that glycans, which serve to decrease the flexibility of the protein chain, can exert a ‘propagated‘ effect on the active site region of the enzyme. This can even alter the electrostatic potential of the heme, and therefore possibly the catalytic properties of the enzyme. Despite this difference in peroxidase activity, the catalytic efficiency of peroxygenase activity toward NBD of artUPO_yeast_ and artUPO_bact_ was more or less equivalent. The *k*_cat_/*K*_M_ values for artUPO enzymes with NBD were tenfold less than that of *Hsp*UPO as a result of a much higher *K*_M_ for this peroxygenase substrate. Interestingly, the *Hsp*UPO that had been expressed in *Pichia* and then deglycosylated, displayed nearly identical kinetics to the glycosylated *Hsp*UPO.^[33]^

### Structure of artUPO

**Figure 2:**
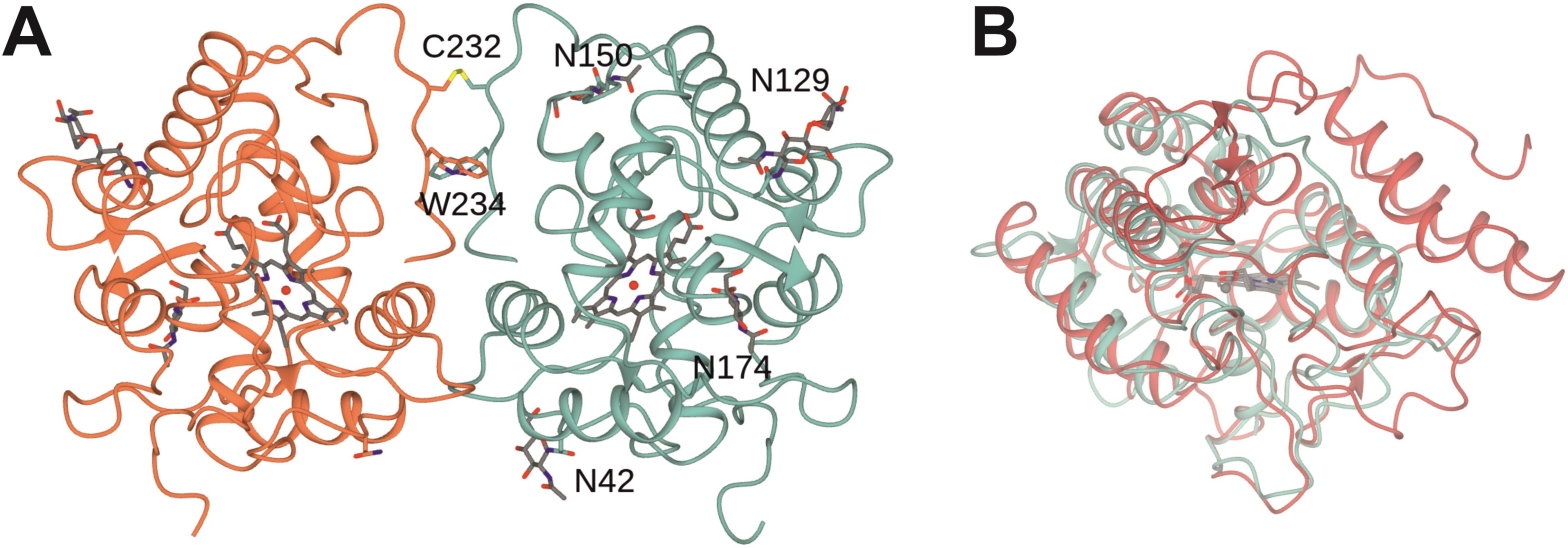
A: Structure of artUPO_yeast_ dimer shown in ribbon format with monomers in coral and blue, and showing interdimer disulphide bridge formed by residues C232. *N*‐Glycosylation sites N42, N129, N150 and N174 in one monomer are also labelled. B: Superimposed structures of monomers of artUPO_yeast_ (blue) and *Aae*UPO Pada‐I mutant (red; PDB code 6EL0).

We first crystallized artUPO_yeast_ and determined the structure of the enzyme in two forms: the first in space group *P*2_1_2_1_2_1_, to a resolution of 2.01 Å and the second in *C*222_1_, to 1.21 Å, with two and one molecules in the asymmetric unit, respectively. The crystallization of artUPO_yeast_ permits a comparison with unpublished structures of the related, native UPO from *Marasmius rotula* that have been deposited by Piontek and co‐workers with PDB accession codes 5FUJ and 5FUK. artUPO_yeast_ is interesting in that, as suggested by analytical size exclusion studies (Figure S8A), it exists as a dimer in solution (Figure 2A), with two monomers connected by a disulfide bridge between C232 on each monomer. Superimposition of the two, non‐equivalent, monomers in the *P*2_1_2_1_2_1_ structure give an rmsd of 0.21 Å over 231 C‐alpha atoms, suggesting that they are very similar. We cannot exclude the possibility of cooperativity in the mechanism of artUPO, although the kinetic plots presented (Figure S7) are not suggestive of more than one phase in ligand binding. The higher resolution structure in the *C*222_1_ space group superimposed with the monomer of the *P*2_1_2_1_2_1_ structure with an rmsd of 0.23 Å over 231 C‐alpha atoms, again indicative of high structural similarity, although clearly the higher resolution presents the enzyme structure in more detail with respect to water structure and alternate conformations of amino acids, which include D61 in the 1.21 Å structure.

The backbone structure of the artUPO_yeast_ monomer (Figure 2B) was compared to known protein structures using the DALI server.^[42]^ *Mro*UPO (5FUJ) was confirmed as the closest structure, with a sequence identity of 73 %, and an rmsd of 0.6 Å over 234 Cα atoms. The next most similar enzyme was the UPO from *Hypoxylon* sp. EC38 (*Hsp*UPO),^[33]^ represented by structure 7O1Z (38 %; 1.4 Å over 225 Cα atoms). The structures confirm that each is a member of the short class of UPOs as defined by Faiza and co‐workers.^[27]^ Interestingly, despite the overall similarity of artUPO to *Hsp*UPO, the latter enzyme does not form a dimer, lacking the extended C‐terminal chain in artUPO that bears both C232 and W234 in artUPO. The next most similar enzymes in the database were *Aae*UPO, represented by its Pada‐I mutant structure 6EL0 (33 %; 2.1 Å over 221 Cα atoms),^[35]^ and chloroperoxidase from *Caldariomyces fumago* (2CIX; 25 %, 1.9 Å over 219).^[43]^ The differences between the structures of artUPO_yeast_ and the long *Aae*UPO‐PaDa‐I are more profound (Figure 2B), the latter displaying what amounts to an extra C‐terminal domain containing two long alpha helices and loops including those between R97 and G114, F204 and E213 and P254 and S272 that are absent or significantly shorter in artUPO_yeast_.

Having been expressed in *Pichia*, artUPO_yeast_ featured glycosylation sites N42, with one resolved *N*‐acetylglucosamine (NAG), N129 (two NAG) and N150 (one NAG), which corresponded to conserved sites N35, N143 and N122 in *Mro*UPO. artUPO_yeast_ however, possessed an additional monoglycosylated site N174 on a shorter loop (M172‐T176) than encountered in 5FUJ (M165‐L171). In addition to an extra glycosylation site, artUPO_yeast_ also possessed W234 in place of alanine in *Mro*UPO (5FUJ) near the C232 residue that participates in the interdimer disulfide bridge. Hydrophobic interactions made by these two tryptophan residues may also contribute to enzyme stability (Figure 2A).

In addition to their overall similarities in fold, artUPO, *Mro*UPO and *Hsp*UPO feature both a glutamic acid and histidine residue (E164 and H93 in artUPO) that forms the catalytic acid‐base pair, in common with CPO and in contrast to the Glu‐Arg dyad found in *Aae*UPO.^[44]^ Significant differences to native *Mro*UPO 5FUJ and *Hsp*UPO were observed in the active site tunnel of artUPO_yeast_ however. *Mro*UPO L149, S156 and G208 are substituted by K156, L163 and N213 in artUPO, respectively, modifications which presumably contribute to altered substrate recognition by artUPO_yeast_, although detailed mutagenesis studies would have to be performed to confirm this. The side chain amine of K156 H‐bonds with the backbone carbonyl groups of G89 and T90 in artUPO_yeast_, and may also confer extra stability to the enzyme. L163 and also F167 in the artUPO tunnel are substituted by G179 and A183 in *Hsp*UPO, which make the active site of artUPO more sterically restricted, and which again may be evidenced by differences in activity between artUPO and *Hsp*UPO (*vide supra*).

**Figure 3:**
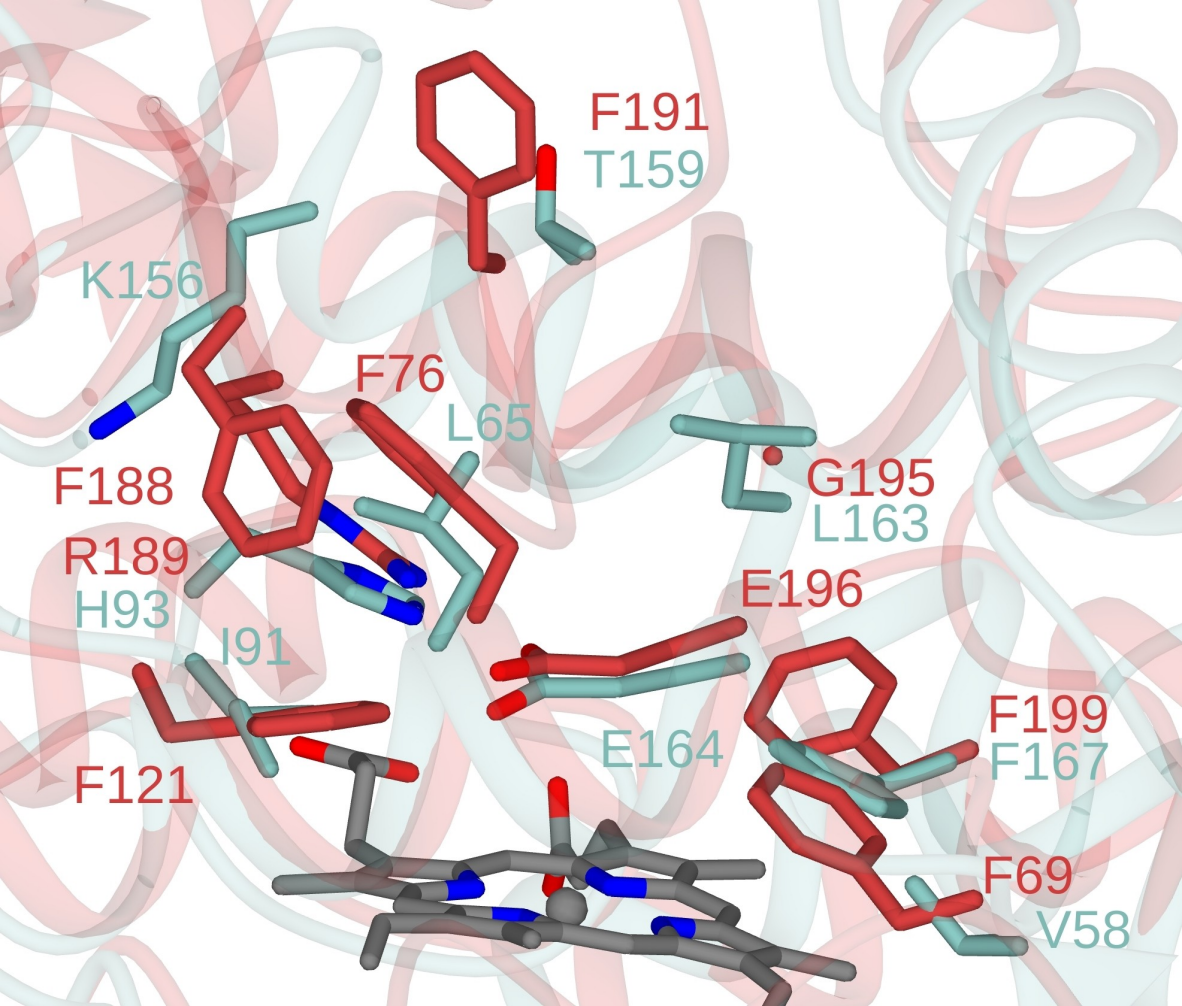
Superimposition of active sites of artUPO_yeast_ and *Aae*UPO PaDa‐I mutant (PDB code 6EL0) with backbone and side chain carbon atoms shown in blue and red, respectively.

The differences between the active site tunnels of artUPO_yeast_ and *Aae*UPO‐PaDa‐I are also noteworthy (Figure 3). artUPO_yeast_ is distinguished from *Aae*UPO by the replacement of characteristic phenylalanine residues in that enzyme with smaller or hydrophilic side chains including I91 for F121, L65 for F76 and K156 for F188, with G195 (common to *Aae*UPO and *Hsp*UPO) replaced by L163 as described above. The differences in active site between *Aae*UPO‐PaDa‐I and *art*UPO give rise to significant variation in substrate range and enantioselectivity, as described below.

The structure of the artUPO expressed in *E. coli* was also determined to see if there were any significant differences that may result from a lack of *in vivo* glycosylation. artUPO_bact_ crystals were obtained in the *P*2_1_2_1_2_1_ space group and again featured two molecules in the asymmetric unit, forming a dimer that was again connected by a disulfide bridge between residues C232. The structure is shown in Figure S9A and a superimposition of artUPO_yeast_ and artUPO_bact_ monomers is shown in Figure S9B. The monomers superimposed with an rmsd of 0.26 A over 232 C alpha atoms, reflective of their very high structural similarity. Few obvious differences could be discerned between the structures of artUPO_yeast_ and artUPO_bact_, except that the N‐terminal residues D6, F7 and S8 could be modelled in the latter, but not the former, and there was of course no glycosylation on asparagine sites N42, N129, N150 and N174 in the bacterially expressed enzyme.

**Figure 4:**
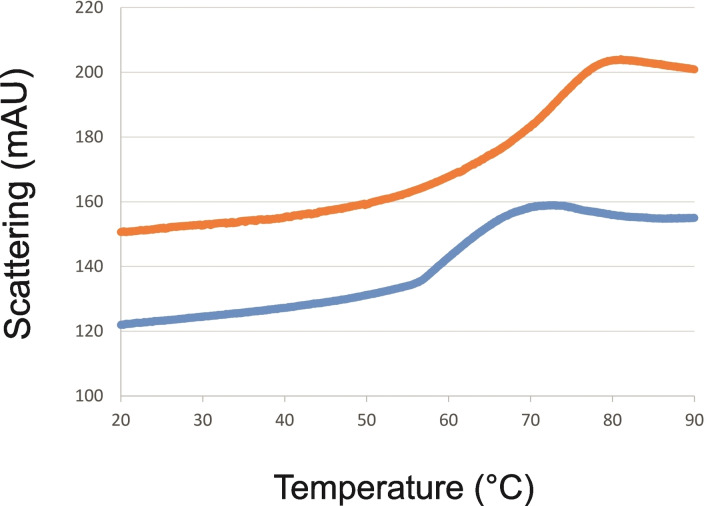
Nano‐DSF analysis of scattering with increasing temperature for artUPO_yeast_ (orange line) and artUPO_bact_ (blue).

Studies on glycosylated and deglycosylated forms of *Hsp*UPO, each expressed in *Pichia*, have suggested that glycosylation may serve to stabilise that UPO to process conditions during biotransformation reactions, although the melting temperature of each enzyme form was reported to be the same.^[33]^ We studied both the melting temperatures (*T*_m_) and onset of aggregation temperatures of artUPO_yeast_ and artUPO_bact_ using nano differential scanning fluorimetry (nano‐DSF). artUPO_bact_ gave a simple profile with a calculated *T*_m_ of 47.8 °C, but artUPO_yeast_ gave a more complex two‐phase profile from which the first *T*_m_ was determined to be 45.5 °C, but the second value could not be determined (Figure S10). Of more relevance are the comparative scattering profiles with increasing temperature for the enzymes, which give an indication of the onset of aggregation or precipitation (Figure 4).

The results clearly show that the onset of aggregation for artUPO_bact_ of approximately 59 °C is significantly lower than that for artUPO_yeast_. This lower stability may be a direct consequence of the lack of glycosylation and was indeed manifested in visible precipitation of artUPO_bact_ in biotransformation reactions (*vide infra*). Poorer process stability was also observed for the deglycosylated form of *Hsp*UPO,^[33]^ although it was not reported that nano‐DSF was used to acquire those data.

### Biotransformations

**Scheme 2:**
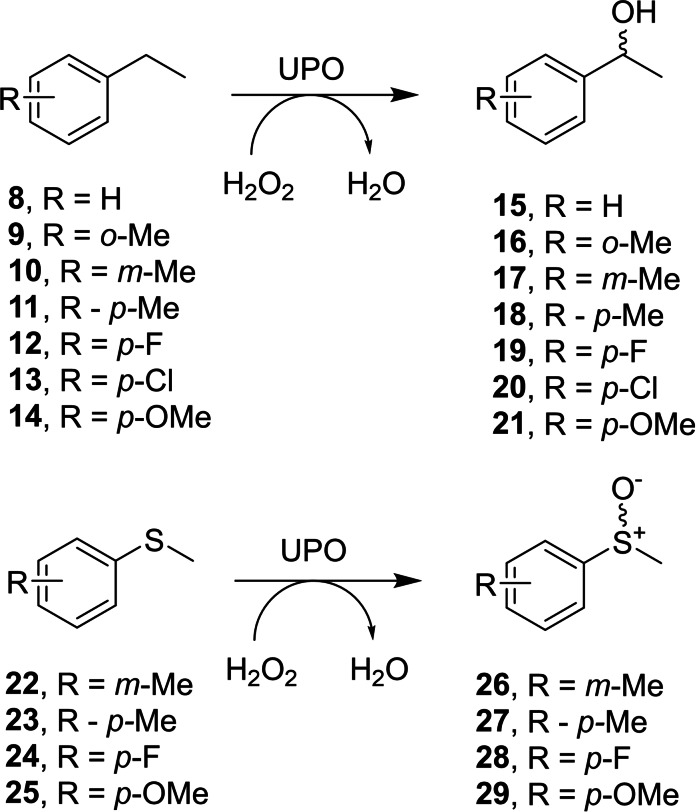
Ethylbenzene and sulfide substrates used in this study.

Following the determination of the structures of artUPO, and their comparison with other UPO structures, we were interested to explore what effects the differences may exert with respect to the biotransformation of standard UPO substrates. We were particularly interested in comparing the activity and enantioselectivity of artUPO_yeast_ with the PaDa‐I mutant of the established *Aae*UPO for simple benzylic hydroxylations^[45]^ and sulfoxidations,[^46^, ^47^] benchmark reactions for UPO oxygenating activity and indicative of the potential utility of the enzymes in the generation of chiral intermediates. Given the poorer stability of the bacterially expressed enzyme, these studies were performed with artUPO_yeast_. Hence the crude supernatant of *Pichia* fermentations expressing either the Pada‐I *Aae*UPO mutant (r*Aae*UPO‐PaDa‐I‐H), expressed and lyophilized as described previously,^[38]^ and artUPO_yeast_ were challenged with a series of ethylbenzene **8**–**14** and sulfide substrates **22**–**25** (Scheme 2) at 10 mM concentration with hydrogen peroxide added at intervals up to 10 mM to optimize conversion. Reactions were monitored by GC to an end point of 6 h and enantiomeric excesses determined by chiral GC analysis (Figures S10 to S20). Longer periods of incubation led to some formation of ketone overoxidation products, so 6 h was chosen as the end‐point to maximise the amount of alcohol product available for chiral analysis in the ethyl benzene series.

Under equivalent reaction conditions r*Aae*UPO‐PaDa‐I‐H gave higher conversions (Table 2), except in the case of *o*‐methyl ethylbenzene **9**. In contrast the *m*‐methyl ethylbenzene **10** was not transformed by artUPO_yeast_. Neither enzyme efficiently transformed ethylbenzene substrates with larger *para‐*chloro (**13**) or *para*‐methoxy (**14**) substituents.

**Table 2:** Conversions and enantiomeric excess of product benzylic alcohols and sulfoxides obtained from UPO‐catalysed oxygenations of ethyl benzene derivatives and sulfide substrates. Enzymes (0.5 U mL^−1^) were challenged with 10 mM substrate in 50 mM PO_4_ buffer with 10 % (w/v) acetonitrile as co‐solvent. Reactions were incubated for 6 h with shaking at 20 °C with additions of 2 mM H_2_O_2_ at 0, 1, 2, 3, 4 and 5 h.

Substrate	r*Aae*UPO‐PaDa‐I‐H		artUPO_yeast_	
	Conv. [%]	ee [%]	Conv. [%]	ee [%]
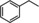		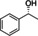		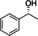
**8**	85	>95 (*R*)‐**15**	38	34 (*R*)‐**15**
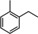		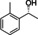		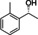
**9**	8	>95 (*R*)‐**16**	38	61 (*R*)‐**16**
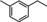		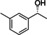		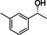
**10**	38	>95 (*R*)‐**17**	<5	n.d.
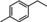		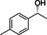		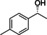
**11**	70	>95 (*R*)‐**18**	35	90 (*R*)‐**18**
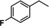		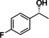		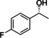
**12**	85	90 (*R*)‐**19**	45	65 (*R*)‐**19**
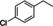		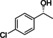		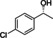
**13**	10	95 (*R*)‐**20**	8	72 (*R*)‐**20**
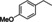		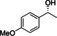		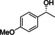
**14**	15	>95 (*R*)‐**21**	5	83 (*R*)‐**21**
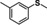		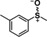		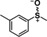
**22**	75	80 *(R/S*)‐**26** ^[a]^	25	23 (*S/R*)‐**26** ^[a]^
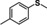		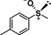		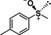
**23**	90	89 (*R*)‐**27**	70	78 (*S*)‐**27**
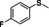		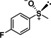		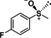
**24**	90	60 (*R*)‐**28**	80	54 (*S*)‐**28**
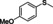		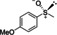		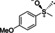
**25**	15	75 (*R*)‐**29** ^[a]^	75	72 (*S*)‐**29**

[a] The absolute configurations of enantiomers of sulfoxide **26** could not be assigned, but the elution order is given, which suggests absolute configurations consistent with other sulfoxidations by *rAae*UPO‐PaDa‐I [(*R*)‐] and artUPO_yeast_ [(*S*)‐]. n.d.=not determined.

The use of the r*Aae*UPO‐PaDa‐I‐H permitted the assignment of absolute configurations of most artUPO reaction products by direct comparison (Table 2) on the basis of precedent oxidations with this and other enzymes (see Supporting Information Section 9 and 10). In all cases (*R*)‐benzyl alcohols were produced by both enzyme systems, although the *ee* values of the products from the r*Aae*UPO‐PaDa‐I‐H reactions were superior in general. This may be a reflection of the greater steric constraints exerted by the larger hydrophobic residues (especially phenylalanines) in the active site of r*Aae*UPO‐PaDa‐I‐H constraining possible poses for hydroxylation at the heme. The 34 % *ee* obtained for the product of hydroxylation (**15**) of ethylbenzene itself (**8**) by artUPO_yeast_, was comparable to that obtained using the short peroxygenase *Hsp*UPO (23 %).^[33]^

A small series of sulfide substrates **22**–**25** was well tolerated by both r*Aae*UPO‐PaDa‐I‐H and artUPO_yeast_. The *meta*‐methyl substrate **22** was transformed more efficiently by *Aae*UPO‐PaDa‐I, as for the ethylbenzene analogue, but artUPO_yeast_ gave a superior conversion for the *para*‐methoxy substrate **25**. However, in contrast to the case with the ethylbenzene substrates, artUPO_yeast_ gave the opposite (*S*)‐ enantiomeric series of sulfoxide products compared to the (*R*)‐ products from r*Aae*UPO‐PaDa‐I‐H observed here and previously by Bassanini and co‐workers.^[46]^ This difference in stereoselective behaviour between r*Aae*UPO‐PaDa‐I‐H and artUPO_yeast_ toward sulfide substrates constitutes a further illustration of the value in exploring UPOs from different classes and organisms for wider selectivity.

Model reactions were also conducted with artUPO_bact_ under conditions used for the biotransformations above. However, it was noticed that the enzyme precipitated upon more prolonged incubations with substrate in the reaction mixture. Despite this, we examined whether the enantioselective properties of artUPO_yeast_ were retained by artUPO_bact_ by using a model substrate as an example. The enzyme was challenged with *para‐*methylthioanisole **23** under similar reaction conditions to those used for artUPO_yeast,_ giving a 65 % conversion to sulfoxide after 2 h. In this case the enantioselectivity was conserved, with the product (*S*)‐**27** having an *ee* of 72 % (Supporting Information Section 10), suggesting that although stability was compromised, enantioselectivity was not significantly affected by the use of the different expression host and the consequent absence of glycosylation in the enzyme.

## Conclusion

The availability of multiple sequences encoding UPOs hints at great potential for unearthing diversity in catalytic characteristics and reaction specificity for application in scalable oxygenations. The accessibility of UPOs will be facilitated by the investigation of complementary expression systems, including both yeast and bacteria, where targets permit. The studies on artUPO in this investigation suggest that catalytic characteristics may be retained upon transferring expression from *P. pastoris* to *E. coli*, although questions remain about enzyme stability in the absence of glycosylation, as highlighted both for this system and also for *Hsp*UPO by Mattevi and co‐workers.^[33]^ However, expression in *E. coli* may facilitate the discovery of improved mutants of short UPOs, which can be optimised and screened for activity and selectivity more quickly in the simpler bacterial system. Once identified, the best mutants could then be transferred for preparative expression of their potentially more stable glycosylated forms in yeast for process applications.

## Experimental Section

**Cloning, expression and purification of artUPO gene in** ***P. pastoris*** **(artUPO_yeast_)**: A gene encoding artUPO was synthesized by GeneArt (ThermoFisher Scientific) with the sequence shown in Figure S2. The gene was amplified from its carrier plasmid using PCR. Using InFusion cloning, the gene was then inserted into a pPICZα vector that had previously been constructed^[38]^ to express the AaeUPO‐PaDa‐I mutant, based on a sequence described in the literature.[^22^, ^23^] In the new construct, the artUPO sequence replaced the coding sequence of *Aae*UPO‐PaDa‐I, but the plasmid retained the mutated signal sequence that had been used to improve expression of that enzyme.[^22^, ^23^] The recombinant plasmid was used to transform into *E. coli* Stellar Cells (TakaRa). The cells were grown overnight on low‐salt LB plates containing 25 μg mL^−1^ zeocin at 37 °C. Five resulting colonies from the transformants were selected for colony PCR and the PCR products were analysed using gel electrophoresis. The colonies that had been successfully transformed were picked and grown in a 10 mL starter culture of low‐salt LB (5 mL) overnight at 37 °C at 180 rpm. The recombinant DNA was extracted using a miniprep kit (Qiagen) and was submitted for sequencing. Following confirmation of the sequence, the recombinant DNA was then linearised with *SacI* and transformed into *Pichia pastoris* strain X‐33 (Invitrogen) *via* electroporation.

A 0.5 L MiniBio fermenter (Applikon) was charged with 200 mL of a basal salts medium containing 26.7 mL^−1^ H_3_PO_4_, 85 % (w/v); 1.17 g L^−1^ CaSO_4_ ⋅ 2H_2_O; 18.2 g L^−1^ K_2_SO_4_; 14.9 g L^−1^ MgSO_4_ ⋅ 7H_2_O; 4.13 g L^−1^ KOH; 40.0 g L^−1^ glycerol) and 4.35 mL L^−1^ of PTM_1_ trace salts containing 6.0 g L^−1^ CuSO_4_ ⋅ 5H_2_O; 0.08 g L^−1^ NaI; 3.0 g L^−1^ MnSO_4_ ⋅ H_2_O; 0.2 g L^−1^ Na_2_MoO_4_ ⋅ 2H_2_O; 0.02 g L^−1^ H_3_BO_3_; 0.5 g L^−1^ CoCl_2_; 20.0 g L^−1^ ZnCl_2_; 65 g L^−1^ FeSO_4_ ⋅ 7H_2_O; 0.2 g L^−1^ Biotin; 5.0 mL L^−1^ H_2_SO_4_). The following parameters were set for the fermentation: temperature at 30 °C, pH 5, stirrer limits set to 200–1750 rpm, airflow at 100 mL min^−1^, DO at 30 %, condenser at 70 %, base pump output of 25 %, and antifoam with pump output of 15 %. The pH was adjusted to 5.0 using 28 % ammonium hydroxide (undiluted).

A glycerol stock of the transformed strain of *P. pastoris* was streaked onto a Yeast extract Peptone Dextrose (YPD) plate with 25 μg mL^−1^ zeocin and incubated at 30 °C for 3 d. Four colonies were selected and grown in 4×5 mL Buffered Glycerol‐complex Medium (BMGY) containing 1 % yeast extract; 2 % peptone; 100 mM potassium phosphate pH 6.0; 1.34 % (w/v) yeast nitrogen base; 4×10^−5^ % biotin and 1 % glycerol for 24 h at 30 °C with shaking at 220 rpm. The four starter cultures were pooled and added to the fermentation vessel and the culture was then grown until all the glycerol had been consumed (approximately 20 h. A 100 % methanol feed with PTM_1_ salts (12 mL L^−1^) was then added at 3.6 mL h^−1^ L^−1^ of initial fermentation volume for 4 h. When the culture had adapted to the methanol feed rate, had a steady DO % and had a fast DO spike after stopping the methanol source, the methanol feed rate was doubled to 7.3 mL h^−1^ L^−1^ of initial fermentation volume. After the culture adapted to the increase in feed rate, this was further increased to 10.9 mL h^−1^ L^−1^ of initial fermentation volume. After 32 h, the culture was harvested and centrifuged at 10,000× g for 20 min to remove the cells. The supernatant was decanted and stored at −80 °C.

**Purification of artUPO_yeast_** : The supernatant was slowly defrosted and dialysed against a salt‐free buffer (50 mM Tris/HCl, pH 8.0) at 4 °C in preparation for anion exchange chromatography. A 5 mL HiTrap Q HP column (GE Healthcare) was equilibrated with 5 column volumes (CV) of the salt‐free buffer before loading the protein sample. The column was washed with 5 CV of the salt‐free buffer, followed by a gradient of NaCl from 0 mM to 300 mM over 20 CV at 2.5 mL min^−1^. Fractions of interest were collected and analysed using SDS PAGE (Figure S4A). Those deemed to contain artUPO of sufficient purity were pooled and the volume of these collected fractions was then reduced to 2 mL using a 10 kDa cut‐off Centricon® filter membrane. The 2 mL of concentrated protein was the loaded onto a HiLoad 16/600 Superdex 75 prep grade column (GE Healthcare) that had been previously equilibrated with a buffer containing 50 mM Tris, 300 mM NaCl and 10 % *w/v* glycerol at pH 8.0. The sample was eluted with 1.2 CV of the Tris buffer at a flow rate of 0.8 mL min^−1^ and the fractions were analysed using SDS PAGE (Figure S4B). Fractions containing pure artUPO were pooled and stored at 4 °C.

**Expression and purification of artUPO gene in** ***E. coli*** **(artUPO_bact_)**: The gene sequence of artUPO was codon optimised and the sequence ordered from GeneArt inserted into pET‐28a(+) (Figure S5). For the expression of artUPO_bact_ in *E. coli*, the intact pET‐28(+) plasmid containing the artUPO gene was used to transform *E. coli* Rosetta pLysS (DE3) cells. Following transformation, a single colony was selected and added to 10 mL LB medium containing 30 μg mL^−1^ kanamycin and 33 μg mL^−1^ chloramphenicol and grown overnight at 37 °C with shaking at 180 rpm. The starter culture was then added to 1 L LB broth in a 2 L shake flask containing kanamycin and chloramphenicol. This culture was incubated at 37 °C with shaking at 180 rpm until the OD_600_ reached 0.7. To induce expression, 1 mL IPTG (1 M), 2 mL 5‐aminlolevulinic acid (5ALA, 0.5 M) and 2 mL FeSO_4_ (0.2 M) were added and the culture was incubated overnight at 16 °C at 180 rpm. The cultures were centrifuged at 5000×*g* for 20 min, the cell pellets were collected and resuspended in 30 mL of 10 mM Tris‐HCl buffer containing 300 mM NaCl, 10 % (v/v) glycerol and 20 mM imidazole. The cells were then lysed using a cell disruptor with a pressure of 26 kPsi and were then centrifuged at 15000×*g* for 40 min. The supernatant was filtered and then loaded onto a 5 mL HisTrap FF crude column (GE Healthcare) that had been equilibrated with 5 column volumes (CV) of cell resuspension buffer. The protein sample was loaded and the column eluted with 5 CV of the buffer, followed by a gradient of 20 mM to 300 mM imidazole over 20 CV. Fractions were collected and analysed using SDS PAGE (Figure S6A). Fractions containing artUPO_bact_ were pooled and concentrated to a volume of 2 mL and then loaded onto a HiLoad 16/600 Superdex 75 prep grade column (GE Healthcare). This column was eluted with 1.2 CV of buffer containing 10 mM Tris‐HCl with 300 mM NaCl, and 10 % (v/v) glycerol at 0.8 mL min‐1 using the FPLC system (ÄKTA Pure or ÄKTA Start, GE Healthcare). Fractions of interest were collected and analysed using SDS PAGE (Figure S6B).

**Determination of kinetic constants**: Kinetic constants for peroxidase and peroxygenase activity for artUPO_yeast_ and artUPO_bact_ were determined in UV assays using the substrates 2,2’‐azino‐bis(3‐ethylbenzthiazoline‐6‐sulfonic acid) (ABTS) and 1,2‐(methylenedioxy‐4‐nitrobenzene) (NBD), respectively. The reactions for both substrates were carried out on a 1 mL scale in 1 mL quartz cuvettes. For the ABTS assays, the reactions contained 50 mM citrate buffer at pH 4.4, ABTS at concentrations of 10, 20, 50, 75, 100, 150, 200, 300, 400 and 500 μM and 1 μL artUPO (from a stock concentration of 4 mg mL^−1^). Reactions were initiated by the addition of H_2_O_2_ to a final concentration of 2 mM. For the NBD assays, the reactions contained 50 mM potassium phosphate buffer at pH 7.0, NBD with final concentrations of 10, 15, 20, 50, 75, 100, 125, 150, 200, 250 and 300 μM in acetonitrile (10 % of the final volume), 1 μL artUPO (from a stock concentration of 4 mg mL^−1^). Reactions were again initiated by the addition of H_2_O_2_ to a final concentration of 2 mM. ABTS and NBD reactions were monitored for 1 min at 418 nm for ABTS and 425 nm for NBD. Monitoring the rate of reaction at different concentrations generated Michaelis‐Menten curves for both ABTS and NBD for artUPO_yeast_ (Figures S7A and S7B, respectively) and artUPO_bact_ (Figures S7C and S7D, respectively).

### Crystallisation

**artUPO_yeas_**_t_: Purified artUPO_yeast_ was concentrated to 60 mg mL^−1^ in 50 mM Tris pH 8.0, 300 mM NaCl and 10 % *w/v* glycerol. Initial crystallisation screens were set up in 96 well 2 drop plates, using a Mosquito robot, which contained either 1 : 1 or 1 : 2 ratio of protein to buffer in a sitting drop with a total volume of 300 nL. The best initial crystals were obtained from conditions containing 0.2 M calcium chloride dihydrate and 20 % (w/v) PEG 3350 at pH 5.1. These gave a dataset in the *P*2_1_2_1_2_1_ space group. Further crystals were obtained in conditions containing 0.15 M KSCN, 25 % PEG MME 2000 with no buffer. These gave a higher resolution dataset in the *C*222_1_ space group.

**artUPO_bac_**_t_: Purified artUPO_bact_ at a concentration of 15 mg mL^−1^ in the size exclusion buffer (10 mM Tris‐HCl with 300 mM NaCl, and 10 % (v/v) glycerol) was similarly subjected to crystal trials. The best crystals were obtained in 0.1 M HEPES pH 7.5 with 25 % (w/v) PEG 3350 and these were harvested without further optimisation for data collection.

**Data collection, structure solution and refinement**: Crystals of artUPO_yeast_ and artUPO_bact_ were flash‐cooled using liquid nitrogen without extra cryo‐protectant. The datasets described in this report were collected at the Diamond Light Source, Didcot, Oxfordshire, U.K. on beamline I03 and I04‐1 (artUPO_yeast_) and beamline I03 (artUPO_bact_). Data were processed and integrated using XDS^[48]^ and scaled using SCALA^[49]^ included in the Xia2 processing system^[50]^ Data collection statistics are provided in Table S1. Crystals of artUPO_yeast_ were obtained in either space group *P*2_1_2_1_2_1_ or *C*222_1_, with two or one molecules in the asymmetric unit, respectively. Crystals of artUPO_bact_ were obtained in the *P*2_1_2_1_2_1_ space group. The solvent content in the *P*2_1_2_1_2_1_ or *C*222_1_ artUPO_yeast_ crystals was 55 % in each case, and 37 % in the case of artUPO_bact_. The structures were solved by molecular replacement using MOLREP^[51]^ with one monomer of *Mro*UPO (73 % sequence identity, PDB code 5FUJ) as the model.

The structures were built and refined using iterative cycles in Coot^[52]^ and REFMAC,^[53]^ employing local NCS restraints in the refinement cycles when appropriate. The final *P*2_1_2_1_2_1_, *C*222_1_ and artUPO_bact_ structures exhibited % *R*_cryst_/*R*_free_ values of 18.2/21.8, 15.0/16.6 and 18.9/23.7, respectively. Refinement statistics for the structures are presented in Table S1. The Ramachandran plot for the *P*2_1_2_1_2_1_ artUPO_yeast_ structure showed 95.0 % of residues to be situated in the most favoured regions, 4.9 % in additional allowed and no residues in outlier regions; the figures for the *C*222_1_ artUPO_yeast_ structure were 95.9 %, 3.7 % and 0.4 %, respectively. The figures for the artUPO_bact_ structure were 99.5 %, 0.5 % and 0.0 %, respectively. Structure factors and coordinate files for artUPO_yeast_ *P*2_1_2_1_2_1_, artUPO_yeast_ *C*222_1_ and artUPO_bact_ have been deposited in the Protein Data Bank (PDB) with the accession codes 7ZNM, 7ZNV and 7ZNW, respectively.

**NanoDSF (nano‐differential scanning fluorimetry)**: artUPO_bact_ and artUPO_yeast_ were prepared at concentrations of 2 mg mL^−1^ in buffer containing 50 mM Tris, 300 mM NaCl pH 8.0. Samples of each were loaded onto a Prometheus NT 48 (NanoTemper) machine in 10 μL capillary tubes. The fluorescence was measured at 330 nm and 350 nm between 20 and 80 °C using a temperature gradient of 1 °C min^−1^. The data were analysed using ThermalControl (NanoTemper).

**Biotransformations**: Oxygenation reactions using *Aae*UPO Pada‐I mutant and artUPO_yeast_ with ethylbenzene substrates **8**–**14** or sulfide substrates **22**–**25** were carried out on a 5 mL scale with 50 mM potassium phosphate buffer pH 7.0, 0.5 U mL^−1^ UPO and 10 mM substrate with 10 % (v/v) acetonitrile. Reactions were incubated with shaking at 250 r.p.m. at 20 °C for 6 h with 5 additions of 2 mM H_2_O_2_ at 0, 1, 2, 3, 4 and 5 h. After 6 h, the reactions were extracted with ethyl acetate and analysed for both conversion % and enantiomeric excess using GC.

**GC analysis**: Conversions were determined using GC analysis with a HP‐5 column (30× 0.25 internal diameter, 0.25 μm film thickness) from Agilent J&W and using helium as a carrier gas. Chiral GC was performed on the same instrument fitted with either a Betadex 120 (Supelco) or BGB 175 (BGB Analytik) chiral column using conditions listed in Table S2. Alcohols **15**, **18**, **19** and **20** were assigned absolute configurations based on elution orders obtained with identical compounds also separated on Supleco Betadex 120 by Decarlini and co‐workers^[54]^ and references therein. Alcohols **16** and **17** were assigned absolute configurations based on elution orders obtained with identical compounds also separated on Supleco Betadex 120 by Li and co‐workers.^[55]^ Sulfoxides **27**, **28** and **29** were assigned absolute configurations based on chiral analysis of biotransformations conducted in house using the cyclohexanone monooxygenase from *Acinetobacter calcoaceticus*,^[56]^ which were compared to results obtained and configurations assigned by Carrea and co‐workers using the same enzyme.^[57]^

## Supporting Information

Gene and amino acid sequences; PCR primers; UV analysis and analytical size exclusion chromatography; kinetics; crystal data; standard and chiral GC analysis conditions are all available in the Supporting Information.

## Conflict of interest

The authors declare no conflict of interest.

1

## Supporting information

As a service to our authors and readers, this journal provides supporting information supplied by the authors. Such materials are peer reviewed and may be re‐organized for online delivery, but are not copy‐edited or typeset. Technical support issues arising from supporting information (other than missing files) should be addressed to the authors.

Supporting Information

## Data Availability

The data that support the findings of this study are available from the corresponding author upon reasonable request.

## References

[1] D. J. Leak , R. A. Sheldon , J. M. Woodley , P. Adlercreutz , Biocatal. Biotransform. 2009, 27, 1–26.

[2] D. Holtmann , M. W. Fraaije , I. W. C. E. Arends , D. J. Opperman , F. Hollmann , Chem. Commun. 2014, 50, 13180–13200. 10.1039/c3cc49747j 24902635

[3] J. Dong , E. Fernández-Fueyo , F. Hollmann , C. E. Paul , M. Pesic , S. Schmidt , Y. Wang , S. Younes , W. Zhang , Angew. Chem. Int. Ed. 2018, 57, 9238–9261; 10.1002/anie.201800343 PMC6099261 29573076

[4] C. Peters , R. M. Buller , Catalysts 2019, 9, 221.

[5] W. J. H. van Berkel , N. M. Kamerbeek , M. W. Fraaije , J. Biotechnol. 2006, 124, 670–689. 16712999 10.1016/j.jbiotec.2006.03.044

[6] R. Fasan , ACS Catal. 2012, 2, 647–666.

[7] J. A. McIntosh , C. C. Farwell , F. H. Arnold , Curr. Opin. Chem. Biol. 2014, 19, 126–134. 24658056 10.1016/j.cbpa.2014.02.001 PMC4008644

[8] R. K. Zhang , X. Huang , F. H. Arnold , Curr. Opin. Chem. Biol. 2019, 49, 67–75. 30343008 10.1016/j.cbpa.2018.10.004 PMC6461521

[9] V. B. Urlacher , M. Girhard , Trends Biotechnol. 2019, 37, 882–897. 30739814 10.1016/j.tibtech.2019.01.001

[10] M. W. Peters , P. Meinhold , A. Glieder , F. H. Arnold , J. Am. Chem. Soc. 2003, 125, 13442–13450. 14583039 10.1021/ja0303790

[11] S. C. Hammer , G. Kubik , E. Watkins , S. Huang , H. Minges , F. H. Arnold , Science 2017, 358, 215. 29026041 10.1126/science.aao1482

[12] O. Shoji , S. Yanagisawa , J. K. Stanfield , K. Suzuki , Z. Cong , H. Sugimoto , Y. Shiro , Y. Watanabe , Angew. Chem. Int. Ed. 2017, 56, 10324–10329; 10.1002/anie.201703461 28544674

[13] C. J. D. Mau , R. Croteau , Phytochem. Rev. 2006, 5, 373.

[14] M. Szaleniec , A. M. Wojtkiewicz , R. Bernhardt , T. Borowski , M. Donova , Appl. Microbiol. Biotechnol. 2018, 102, 8153–8171. 30032434 10.1007/s00253-018-9239-3 PMC6153880

[15] J. K. Kulig , C. Spandolf , R. Hyde , A. C. Ruzzini , L. D. Eltis , G. Grönberg , M. A. Hayes , G. Grogan , Bioorg. Med. Chem. 2015, 23, 5603–5609. 26234905 10.1016/j.bmc.2015.07.025

[16] C. J. C. Whitehouse , S. G. Bell , L.-L. Wong , Chem. Soc. Rev. 2012, 41, 1218–1260. 22008827 10.1039/c1cs15192d

[17] S. Bormann , A. Gomez Baraibar , Y. Ni , D. Holtmann , F. Hollmann , Catal. Sci. Technol. 2015, 5, 2038–2052.

[18] Y. Wang , D. Lan , R. Durrani , F. Hollmann , Curr. Opin. Chem. Biol. 2017, 37, 1–9. 27992798 10.1016/j.cbpa.2016.10.007

[19] R. Ullrich , J. Nüske , K. Scheibner , J. Spantzel , M. Hofrichter , Appl. Environ. Microbiol. 2004, 70, 4575. 15294788 10.1128/AEM.70.8.4575-4581.2004 PMC492325

[20] R. Ullrich , M. Hofrichter , FEBS Lett. 2005, 579, 6247–6250. 16253244 10.1016/j.febslet.2005.10.014

[21] M. J. Pecyna , R. Ullrich , B. Bittner , A. Clemens , K. Scheibner , R. Schubert , M. Hofrichter , Appl. Microbiol. Biotechnol. 2009, 84, 885–897. 19434406 10.1007/s00253-009-2000-1

[22] P. Molina-Espeja , E. Garcia-Ruiz , D. Gonzalez-Perez , R. Ullrich , M. Hofrichter , M. Alcalde , Appl. Environ. Microbiol. 2014, 80, 3496–3507. 24682297 10.1128/AEM.00490-14 PMC4018863

[23] P. Molina-Espeja , S. Ma , D. M. Mate , R. Ludwig , M. Alcalde , Enzyme Microb. Technol. 2015, 73–74, 29–33. 10.1016/j.enzmictec.2015.03.004 26002501

[24] S. Peter , M. Kinne , X. Wang , R. Ullrich , G. Kayser , J. T. Groves , M. Hofrichter , FEBS J. 2011, 278, 3667–3675. 21812933 10.1111/j.1742-4658.2011.08285.x PMC3586278

[25] M. Kluge , R. Ullrich , C. Dolge , K. Schibner , M. Hofrichter , Appl. Microbiol. Biotechnol. 2009, 81, 1071–1076. 18815784 10.1007/s00253-008-1704-y

[26] M. Kinne , M. Poraj-Kobielska , E. Aranda , R. Ullrich , K. E. Hammel , K. Scheibner , M. Hofrichter , Bioorg. Med. Chem. Lett. 2009, 19, 3085–3087. 19394224 10.1016/j.bmcl.2009.04.015

[27] M. Faiza , S. Huang , D. Lan , Y. Wang , BMC Evol. Biol. 2019, 19, 76. 30866798 10.1186/s12862-019-1394-3 PMC6417270

[28] E. D. Babot , J.C. del Rio , L. Kalum , A. T. Martínez , A. Gutiérrez , Biotechnol. Bioeng. 2012, 110, 2323–2332. 10.1002/bit.24904 23519689

[29] D. H. Anh , R. Ullrich , D. Benndorf , A, Svatoś , A. Muck , M. Hofrichter , Appl. Environ. Microbiol. 2007, 7, 5477–5485. 10.1128/AEM.00026-07 PMC2042081 17601809

[30] A. Knorrscheidt , J. Soler , N. Hünecker , P. Püllmann , M. Garcia-Borràs , M. J. Weissenborn , ACS Catal. 2021, 11, 7327–7338. 34631225 10.1021/acscatal.1c00847 PMC8496131

[31] P. Püllmann , A. Knorrscheidt , J. Münch , P. R. Palme , W. Hoehenwarter , S. Marillonnet , M. Alcalde , M. J. Weissenborn , Commun. Biol. 2021, 4, 562. 33980981 10.1038/s42003-021-02076-3 PMC8115255

[32] G. Gröbe , R. Ullrich , M. J. Pecyna , D. Kapturska , S. Friedrich , M. Hofrichter , K. Scheibner , AMB Express 2011, 1, 31. 21988939 10.1186/2191-0855-1-31 PMC3214178

[33] L. Rotilio , A. Swoboda , K. Ebner , C. Rinnofner , A. Glieder , W. Kroutil , A. Mattevi , ACS Catal. 2021, 11, 11511–11525. 34540338 10.1021/acscatal.1c03065 PMC7611678

[34] K. Piontek , E. Strittmatter , R. Ullrich , G. Gröbe , M. J. Pecyna , M. Kluge , K. Scheibner , M. Hofrichter , D. A. Plattner , J. Biol. Chem. 2013, 288, 34767–34776. 24126915 10.1074/jbc.M113.514521 PMC3843090

[35] M. Ramirez-Escudero , P. Molina-Espeja , P. Gomez de Santos , M. Hofrichter , J. Sanz-Aparicio , M. Alcalde , ACS Chem. Biol. 2018, 13, 3259–3268. 30376293 10.1021/acschembio.8b00500

[36] J. Carro , A. González-Benjumea , E. Fernández-Fueyo , C. Aranda , V. Guallar , A. Gutiérrez , A. T. Martínez , ACS Catal. 2019, 9, 6234–6242.

[37] J. Vind, L. Kiemer, E. Amourgi, **2016** WO 2016207373A1.

[38] H. E. Bonfield , K. Mercer , A. Diaz-Rodriguez , G. C. Cook , B. S. J. McKay , P. Slade , G. M. Taylor , W. X. Ooi , J. D. Williams , J. P. M. Roberts , J. A. Murphy , L. Schmermund , W. Kroutil , T. Mielke , J. Cartwright , G. Grogan , L. J. Edwards , ChemPhotoChem 2020, 4, 45–51.

[39] S. M. Hoffmann , H.-R. Danesh-Azari , C. Spandolf , M. J. Weissenborn , G. Grogan , B. Hauer , ChemCatChem 2016, 8, 3234–3239.

[40] E. Fernández Fueyo, C. Aranda Oliden, A. Gutiérrez Suárez, A. T. Martínez Ferrer, **2020**, EP3594332A1.

[41] S. Škulj , A. Barišić , N. Mutter , O. Spadiut , I. Barišić , B. Bertoša , Comput. Struct. Biotechnol. J. 2022, 20, 3096–3105. 35782731 10.1016/j.csbj.2022.06.008 PMC9233188

[42] L. Holm , Methods Mol. Biol. 2020, 2112, 29–42. 32006276 10.1007/978-1-0716-0270-6_3

[43] K. Kuhnel , W. Blankenfeldt , J. Terner , I. Schlichting , J. Biol. Chem. 2006, 281, 23990–23998. 16790441 10.1074/jbc.M603166200

[44] X. Wang , R. Ullrich , M. Hofrichter , J. T. Groves , Proc. Natl. Acad. Sci. USA 2015, 112, 3686–3691. 25759437 10.1073/pnas.1503340112 PMC4378415

[45] M. Kluge , R. Ullrich , K. Scheibner , M. Hofrichter , Green Chem. 2012, 14, 440–446.

[46] I. Bassanini , E. E. Ferrandi , M. Vanoni , G. Ottolina , S. Riva , M. Crotti , E. Brenna , D. Monti , Eur. J. Org. Chem. 2017, 2017, 7186–7189.

[47] Y. Li , Y. Ma , P. Li , X. Zhang , D. Ribitsch , M. Alcalde , F. Hollmann , Y. Wang , ChemPlusChem 2020, 85, 254–257. 31951316 10.1002/cplu.201900751

[48] W. Kabsch , Acta Crystallogr. Sect. D. 2010, 66, 125–132. 20124692 10.1107/S0907444909047337 PMC2815665

[49] P. Evans , Acta Crystallogr. Sect. D. 2006, 62, 72–82. 16369096 10.1107/S0907444905036693

[50] G. Winter , J. Appl. Crystallogr. 2010, 3, 186–190.

[51] A. Vagin , A. Teplyakov , J. Appl. Crystallogr. 1997, 30, 1022–1025.

[52] P. Emsley , K. Cowtan , Acta Crystallogr. Sect. D 2004, 60, 2126–2132. 15572765 10.1107/S0907444904019158

[53] G. N. Murshudov , A. A. Vagin , E. J. Dodson , Acta Crystallogr. Sect. D 1997, 53, 240–255. 15299926 10.1107/S0907444996012255

[54] M. F. Decarlini , M. L. AImar , A. M. Vàzquez , S. Vero , L. I. Rossi , P. Yang , Biocatal. Agric. Biotechnol. 2017, 12, 275–285.

[55] W. Li , G. Hou , C. Wang , Y. Jiang , X. Zhang , Chem. Commun. 2010, 46, 3979–3981. 10.1039/b927028k 20407691

[56] B. D. Summers, **2014**, PhD thesis University of York.

[57] G. Carrea , B. Redigolo , S. Riva , S. Colonna , N. Gaggero , E. Battistel , D. Bianchi , Tetrahedron: Asymmetry 1992, 3, 1063–1068.

