# Supplementary material for: Comparing the Catalytic and Structural Characteristics of a ‘Short’ Unspecific Peroxygenase (UPO) Expressed in Pichia pastoris and Escherichia coli
Source: Chembiochem. 2022 Nov 30;24(1):e202200558. doi: 10.1002/cbic.202200558 (PMC10098773; doi:10.1002/cbic.202200558)
Supplement: Supplementary file 1 — Supporting Information [file CBIC-24-0-s001.pdf]

ChemBioChem

Supporting Information

# Comparing the Catalytic and Structural Characteristics of a 'Short' Unspecific Peroxygenase (UPO) Expressed in *Pichia pastoris* and *Escherichia coli*

Wendy X. Q. Robinson, Tamara Mielke, Benjamin Melling, Anibal Cuetos, Alison Parkin, William P. Unsworth, Jared Cartwright, and Gideon Grogan*

## 1. Target Selection

```
                    10        20        30        40        50        60
                     |         |         |         |         |         |
artUPO      ------------SQDIVDFSQHPWKAPGPNDLRSPCPGLNTLANHGFLPRNGRNITIPMI
MroUPO      -------------------SAHPWKAPGPNDSRGPCPGLNTLANHGFLPRNGRNISVPMI
AaeUPO      EPGLPPGPLENSSAKLVNDEAHPWKPLRPGDIRGPCPGLNTLASHGYLPRNG-VATPAQI
                               . ****.  *.* *.*********.**:*****   : . *

                    70        80        90       100       110       120
                     |         |         |         |         |         |
artUPO      VQAGFDGYNVQ-------------------PDILILAAKVGLLTSPEPDTFTLDDLKLHG
MroUPO      VKAGFEGYNVQ-------------------SDILILAGKIGMLTSREADTISLEDLKLHG
AaeUPO      INAVQEGFNFDNQAAIFATYAAHLVDGNLITDLLSIGRKTRLTGPDPPPPASVGGLNEHG
            ::*  :*:*.:                   .*:* :. *  :  .  . . :: .*: **

                   130       140       150       160       170       180
                     |         |         |         |         |         |
artUPO      TIEHDASLSREDFALGDNLHFNEAIFNTLANSNP--GSDVYNITSAGQVLKDRLADSLAR
MroUPO      TIEHDASLSREDVAIGDNLHFNEAIFTTLANSNP--GADVYNISSAAQVQHDRLADSLAR
AaeUPO      TFEGDASMTRGDAFFGNNHDFNETLFEQLVDYSNRFGGGKYNLTVAGELRFKRIQDSIAT
            *:* ***::* *  :*:* .***::*  *.: .   *.. **:: *.::  .*: **:*

                   190       200       210       220       230       240
                     |         |         |         |         |         |
artUPO      NPNVTNTGKEFTIRTLESAFYLSVM--GNATTGEAPKNFVQIFFREERLP---IEEGWKR
MroUPO      NPNVTNTDLTATIRSSESAFFLTVMSAGDPLRGEAPKKFVNVFFREERMP---IKEGWKR
AaeUPO      NPNFSFVDFRFFTAYGETTFPANLFVDGRRDDGQLDMDAARSFFQFSRMPDDFFRAPSPR
            ***.: ..        *::*  .::  *    *:   . .. **: .*:*   :.    *

                   250       260       270       280       290       300
                     |         |         |         |         |         |
artUPO      STT------------------PITSDTLNPIAGQIS------------EASNWKPNPDQC
MroUPO      STT------------------PITIPLLGPIIERIT------------ELSDWKPTGDNC
AaeUPO      SGTGVEVVVQAHPMQPGRNVGKINSYTVDPTSSDFSTPCLMYEKFVNITVKSLYPNPTVQ
            * *                   *.   :.*    ::              ..  *.

                   310       320
                     |         |
artUPO      PWIVLSPNL--------------------
MroUPO      GAIVLSPEL--------------------
AaeUPO      LRKALNTNLDFLFQGVAAGCTQVFPYGRD
               .*..:*
```

**Figure S1**. Sequence Alignment of artUPO, *Mro*UPO (derived from PDB deposition code 5FUJ) and *Aae*UPO PaDa-I mutant (AaeUPO, derived from PDB deposition code 6EL0).

## 2. Cloning, Expression and Purification of artUPO Gene in *P. pastoris* (artUPO$_{yeast}$).

```
>artUPO

TCCCAGGATATTGTGGACTTCTCCCAGCATCCGTGGAAGGCACCTGGACCCAACGATCTCCG
CTCCCCCTGTCCCGGACTCAACACGCTCGCGAACCACGGCTTCCTCCCTCGCAACGGTCGAA
ACATCACGATCCCGATGATCGTCCAGGCAGGCTTCGACGGCTACAACGTCCAGCCGGATATC
CTCATTTTGGCAGCCAAGGTGGGATTGCTCACCTCGCCTGAACCCGATACCTTCACCCTCGA
CGATTTGAAGTTGCATGGAACCATCGAGCATGATGCATCGTTGTCGAGGGAGGACTTCGCGC
TCGGCGATAACCTCCACTTCAACGAGGCGATCTTCAACACCCTCGCAAACTCGAACCCTGGT
TCCGATGTCTACAACATCACCTCGGCAGGACAGGTGCTCAAGGACCGCCTCGCCGACTCGCT
CGCCAGGAACCCGAACGTCACGAACACCGGCAAAGAGTTCACTATCAGGACTTTGGAGTCCG
CCTTCTATCTCTCCGTCATGGGCAACGCAACAACAGGTGAAGCACCCAAAAACTTCGTGCAG
ATTTTCTTCCGAGAGGAGCGGCTCCCCATCGAGGAGGGCTGGAAGAGGTCCACAACCCCTAT
CACGTCGGACACGTTGAACCCCATTGCAGGCCAGATTTCGGAGGCGTCGAACTGGAAGCCCA
ACCCCGACCAGTGTCCCTGGATCGTCCTCTCGCCCAACTTG
```

**Figure S2**. Gene encoding artUPO for expression in *Pichia pastoris.*

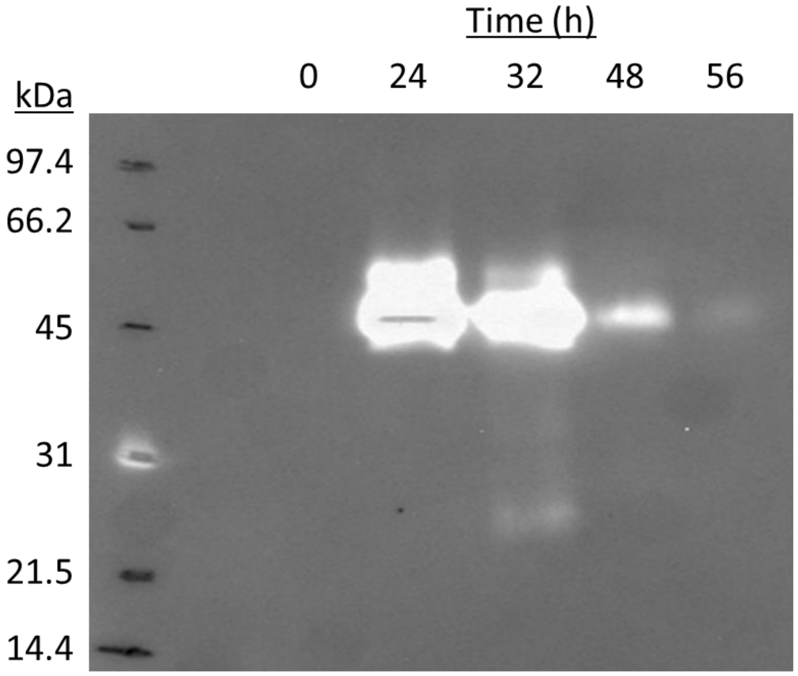


**Figure S3**. Expression of artUPO$_{yeast}$ in *Pichia pastoris* sampled at intervals and analysed by Western blot against anti-His as described above in section **S2**.

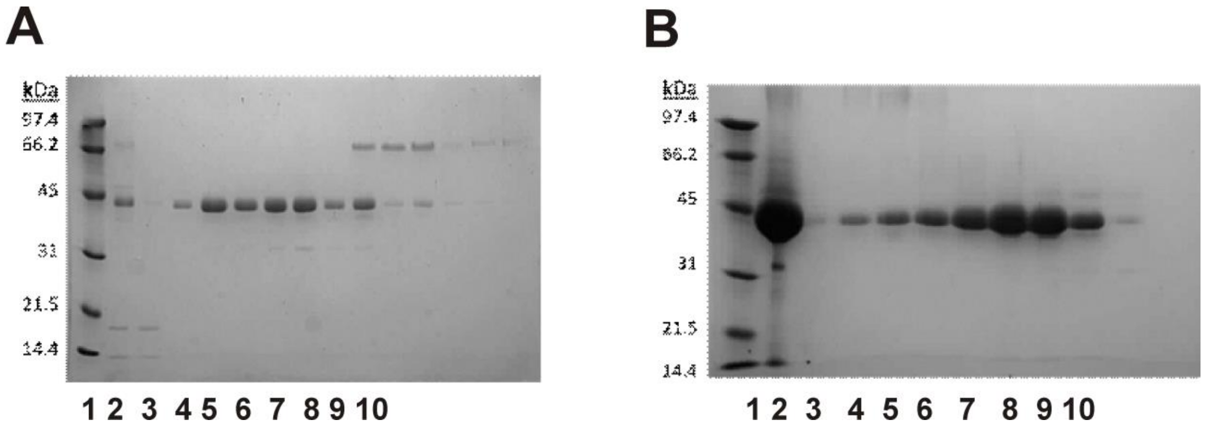


**Figure S4**. Purification of artUPO$_{yeast}$. **A**: SDS-PAGE analysis of fractions from MonoQ anion exchange column of artUPO. Lane 1 – low MW markers; 2: loaded protein sample; 3-10 fractions of MonoQ column; **B**: SDS-PAGE analysis of fractions from size exclusion chromatography of artUPO using a 16/600 Superdex 75 column. Lane 1 – low MW markers; 2: loaded protein sample; 3-10 fractions of SEC column.

### 3. Expression and Purification of artUPO Gene in *E. coli* (artUPO$_{bact}$).

The gene sequence of artUPO was codon optimised and the following sequence ordered from GeneArt inserted into pET-28a(+) (**Figure S5**).

```
ATGAGCCAGGACATCGTGGATTTTAGCCAACATCCGTGGAAAGCGCCGGGTCCGAACGACCT
GCGTAGCCCGTGCCCGGGTCTGAACACCCTGGCGAACCACGGTTTCCTGCCGCGTAACGGCC
GTAACATCACCATTCCGATGATCGTGCAGGCGGGTTTTGACGGCTACAACGTTCAACCGGAT
ATCCTGATTCTGGCGGCGAAGGTTGGTCTGCTGACCAGCCCGGAGCCGGACACCTTCACCCT
GGACGATCTGAAACTGCACGGTACCATTGAGCACGATGCGAGCCTGAGCCGTGAAGACTTTG
CGCTGGGCGATAACCTGCACTTCAACGAAGCGATCTTTAACACCCTGGCGAACAGCAACCCG
GGTAGCGATGTGTACAACATTACCAGCGCGGGCCAGGTTCTGAAGGACCGTCTGGCGGATAG
CCTGGCGCGTAACCCGAACGTGACCAACACCGGCAAAGAGTTCACCATTCGTACCCTGGAAA
GCGCGTTTTATCTGAGCGTGATGGGTAACGCGACCACCGGTGAAGCGCCGAAGAACTTTGTT
CAAATCTTCTTTCGTGAGGAACGTCTGCCGATTGAGGAAGGTTGGAAACGTAGCACCACCCC
GATCACCAGCGACACCCTGAACCCGATTGCGGGTCAGATTAGCGAAGCGAGCAACTGGAAAC
CGAACCCGGATCAATGCCCGTGGATTGTTCTGAGCCCGAACCTGTAA
```

**Figure S5**. Gene encoding artUPO codon optimized for expression in *E. coli.*

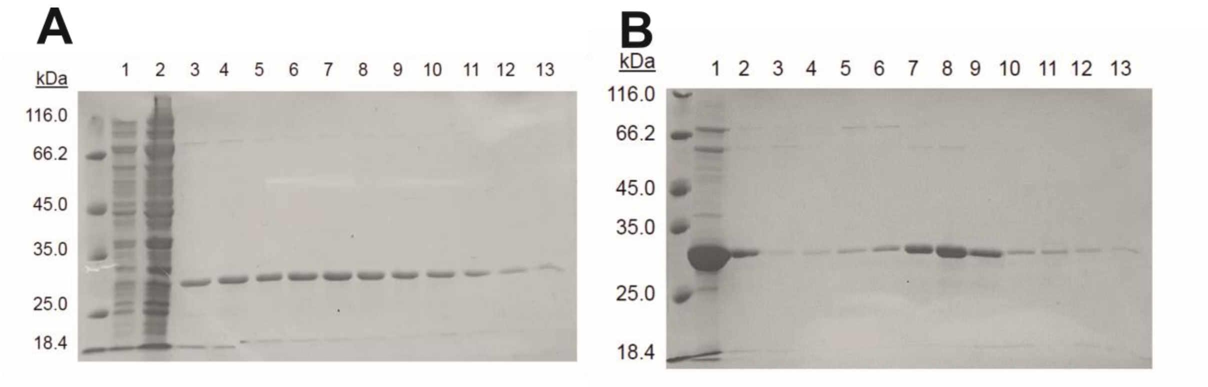


**Figure S6**. **A**: Fractions of NiNTA column used to isolate artUPO$_{bact}$. Lanes 1 and 2 correspond to flow through fractions; Lanes 3-13 correspond to successive fractions analysed over the $A_{280}$ peak; **B**: Fractions of SEC column used to isolate artUPO$_{bact}$.; Lanes 1-13 correspond to successive fractions analysed over the $A_{280}$ peak.

## 4. Determination of Kinetic Constants

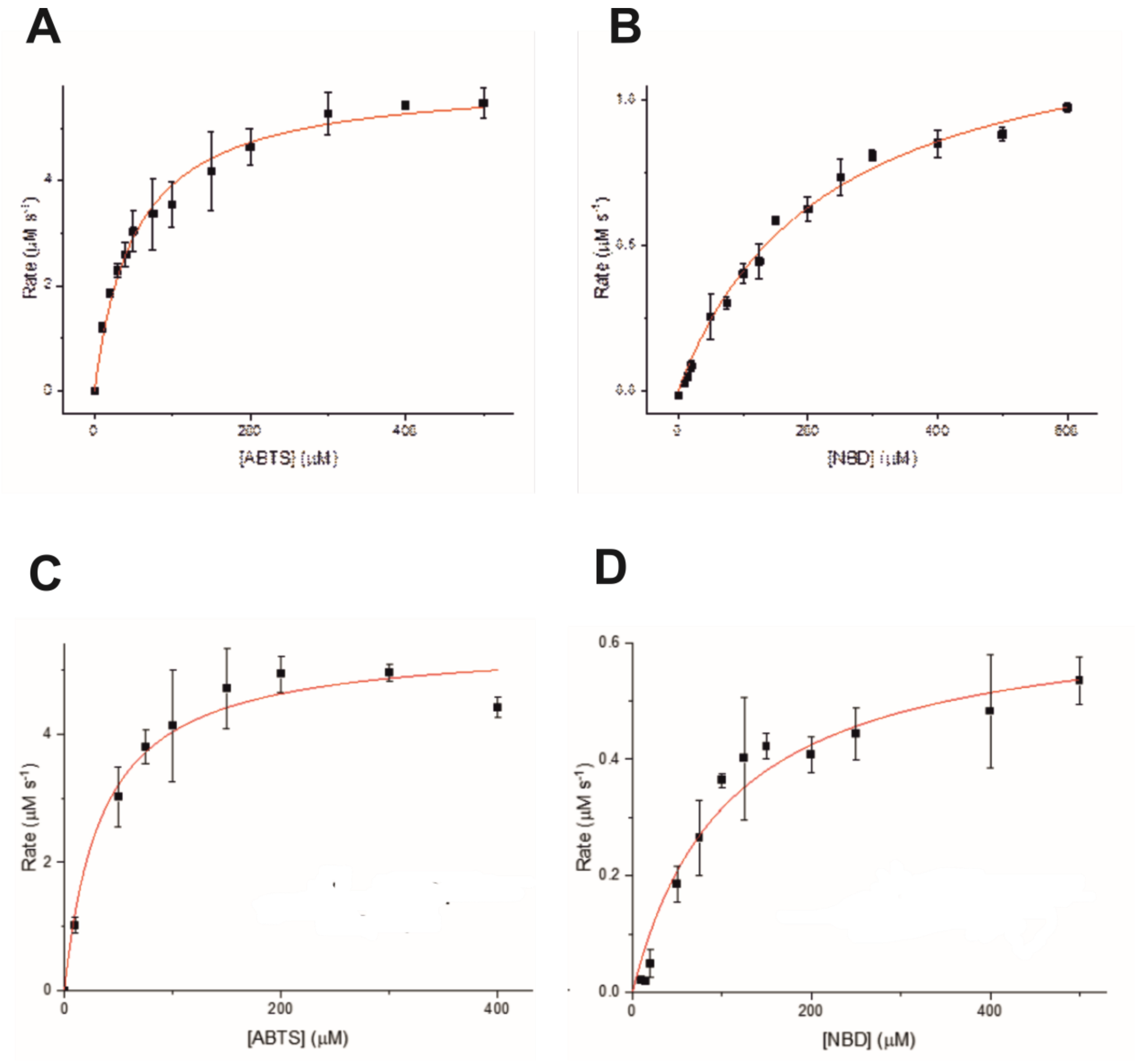


**Figure S7**. Determination of kinetic constants for peroxidase and peroxygenase activity of artUPO$_{yeast}$ with **A**: ABTS and **B**: NBD as substrates and artUPO$_{bact}$ with **C**: ABTS and **D**: NBD as substrates Kinetic constants can be found in manuscript **Table 1**.

## 5. Analytical Size Exclusion Experiments

The oligomeric nature of native artUPO$_{yeast}$ and artUPO$_{bact}$ was compared using analytical size exclusion on a Superdex™ 200 Increase 10/300 GL column using 200 μL of enzyme at 1 mg mL$^{-1}$ in 10 mM potassium phosphate pH 7.4, containing 150 mM NaCl at a flow rate of 0.75 mL min$^{-1}$. Elution profiles were compared to a protein

standard mix (69385 Protein Standard Mix 15, Sigma Aldrich) run on the same column in the same conditions (**Figure S8**).

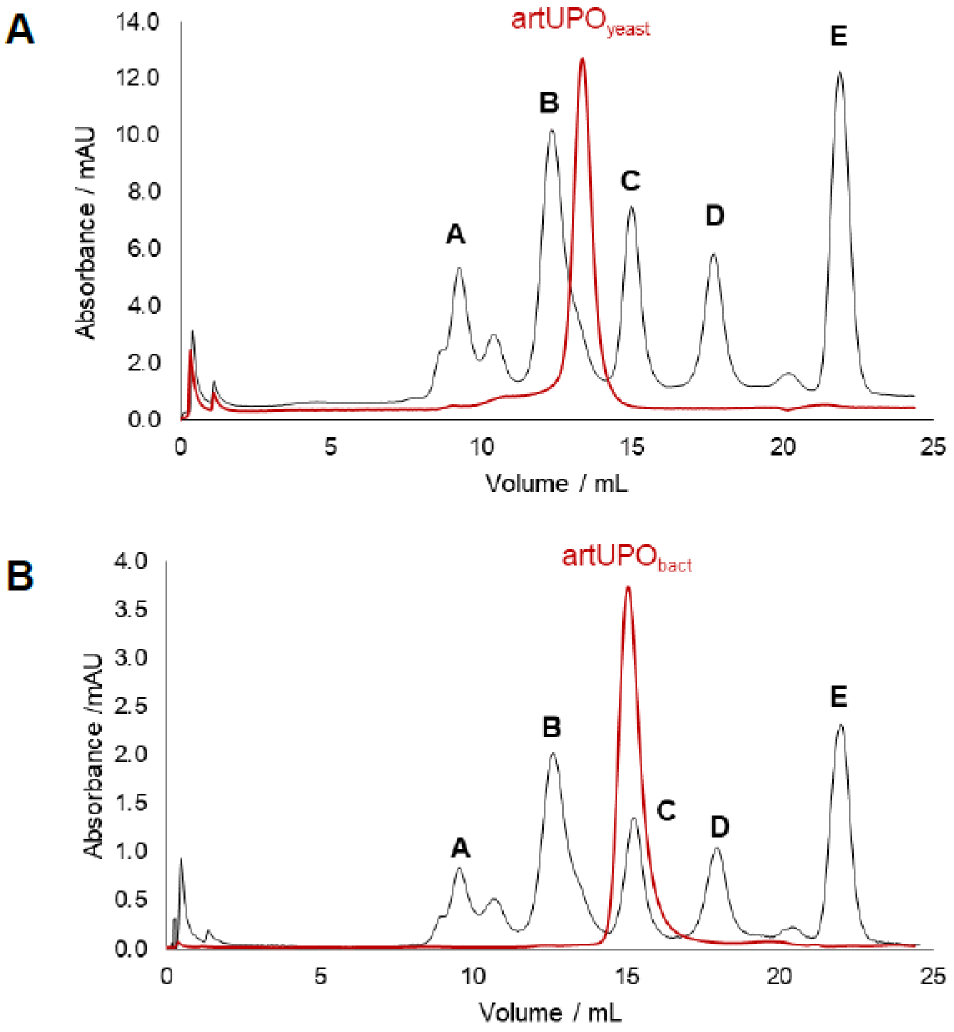


**Figure S8**. Analytical SEC chromatograms for **A**. artUPO$_{yeast}$ and **B**. artUPO$_{bact}$. (red traces). Standards (black trace) are: **A:** Thyroglobulin 670.0 kDa; **B**: γ-globulins from bovine blood 150.0 kDa; **C**: Albumin chicken egg grade VI (ovalbumin) 44.3 kDa; **D**: Ribonuclease A type I-A from bovine pancreas 13.7 kDa; **E**: *p*-aminobenzoic acid (pABA).

## 6. Crystallisation

### artUPO$_{yeast}$

Purified artUPO$_{yeast}$ was concentrated to 60 mg mL$^{-1}$ in 50 mM Tris pH 8.0, 300 mM NaCl and 10% *w/v* glycerol. Initial crystallisation screens were set up in 96 well 2 drop

plates, using a Mosquito robot, which contained either 1:1 or 1:2 ratio of protein to buffer in a sitting drop with a total volume of 300 nL. The best initial crystals were obtained from conditions containing 0.2 M calcium chloride dihydrate and 20% (w/v) PEG 3350 at pH 5.1. These gave a dataset in the $P2_12_12_1$ space group. Further crystals were obtained in conditions containing 0.15 M KSCN, 25% PEG MME 2000 with no buffer. These gave a higher resolution dataset in the $C222_1$ space group.

**artUPO$_{bact}$**

Purified artUPO$_{bact}$ at a concentration of 15 mg mL$^{-1}$ in the size exclusion buffer (10 mM Tris-HCl with 300 mM NaCl, and 10% (v/v) glycerol) was similarly subjected to crystal trials. The best crystals were obtained in 0.1 M HEPES pH 7.5 with 25% (w/v) PEG 3350 and these were harvested without further optimisation for data collection.

## 7. Data collection, structure solution and refinement

**Table S1.** Data Collection and Refinement Statistics for artUPO. Numbers in brackets refer to data for highest resolution shells.

| | **artUPO$_{yeast}$; $P2_12_12_1$** | **artUPO$_{yeast}$; $C222_1$** | **artUPO$_{bact}$** |
|---|---|---|---|
| Beamline | Diamond I03 | Diamond I04-1 | Diamond I03 |
| Wavelength (Å) | 0.976254 | 0.915870 | 0.976230 |
| Resolution (Å) | 50.57-2.01 (2.06-2.01) | 77.27-1.21 (1.23-1.21) | 39.34-2.09 (2.15-2.09) |
| Space Group | $P\ 2_12_12_1$ | $C\ 2\ 2\ 2_1$ | $P\ 2_12_12_1$ |
| Unit cell (Å) | a = 50.34; b = 75.82; c = 151.70<br>$\alpha = \beta = \gamma = 90.00$ | A = 50.85; b = 74.17; c = 154.54<br>$\alpha = \beta = \gamma = 90.00$ | A = 46.03; b = 58.81; c = 151.46<br>$\alpha = \beta = \gamma = 90.00$ |
| No. of molecules in the asymmetric unit | 2 | 1 | 2 |
| Unique reflections | 39554 (2861) | 89172 (4348) | 25200 (1910) |
| Completeness (%) | 99.9 (99.9) | 99.9 (100.0) | 100.0 (100.0) |
| $R_{merge}$ (%) | 0.08 (0.50) | 0.04 (0.43) | 0.12 (0.77) |
| $R_{p.i.m.}$ | 0.04 (0.28) | 0.02 (0.28) | 0.05 (0.32) |
| Multiplicity | 8.0 (7.8) | 7.7 (6.3) | 11.4 (12.7) |
| $<I/\sigma(I)>$ | 16.5 (4.4) | 23.6 (3.8) | 12.8 (3.1) |
| Overall *B* factor from Wilson plot (Å$^2$) | 20 | 11 | 28 |
| $CC_{1/2}$ | 1.00 (0.96) | 1.00 (0.92) | 1.00 (0.94) |
| $R_{cryst}$/ $R_{free}$ (%) | 19.0/22.6 | 15.0/16.6 | 18.9/23.7 |
| r.m.s.d 1-2 bonds (Å) | 0.009 | 0.019 | 0.008 |
| r.m.s.d 1-3 angles (°) | 1.62 | 2.18 | 1.51 |
| Avge main chain B (Å$^2$) | 29 | 13 | 32 |
| Avge side chain B (Å$^2$) | 32 | 16 | 34 |
| Avge water B (Å$^2$) | 34 | 17 | 31 |

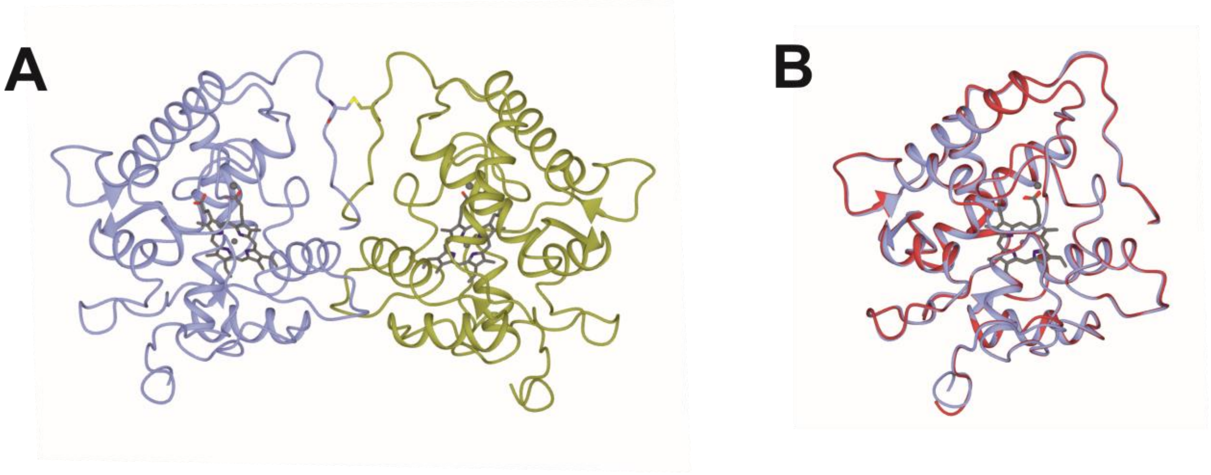


**Figure S9**. **A**: Structure of $artUPO_{bact}$ dimer with monomers shown in blue and gold; **B**: Superimposition of monomers of $artUPO_{bact}$ and $artUPO_{yeast}$ shown in blue and red respectively.

**8. NanoDSF (Nano Differential Scanning Fluorimetry)**

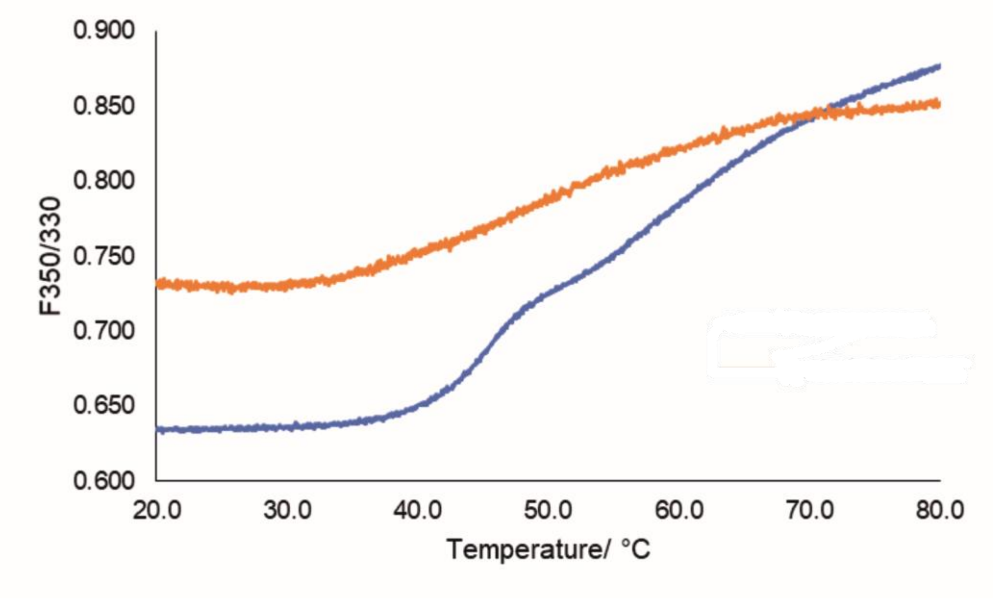


**Figure S10**. Nano-DSF analysis of $artUPO_{yeast}$ (blue line) and $artUPO_{bact}$ (orange). The ratio of the fluorescence at 350 nm and 330 nm was measured against a temperature gradient of 20–80 °C using 2 mg $mL^{-1}$ of each enzyme.

## 9. GC Analysis

**Table S2**. Conditions for chiral separations of oxygenation products.

| Compound | Column | Conditions | Retention Times of Enantiomers (min) |
|---|---|---|---|
| **15** | Betadex 120 | 120 °C isothermal | 6.65 and 7.07 |
| **16** | Betadex 120 | 120 °C isothermal | 12.00 and 13.24 |
| **17** | Betadex 120 | 120 °C isothermal | 10.40 and 10.76 |
| **18** | Betadex 120 | 120 °C isothermal | 9.94 and 10.40 |
| **19** | Betadex 120 | 120 °C isothermal | 7.35 and 7.71 |
| **20** | Betadex 120 | 120 °C isothermal | 22.18 and 23.75 |
| **21** | Betadex 120 | 120 °C isothermal | 28.36 and 27.40 |
| **26** | BGB 175 | 180 °C isothermal | 5.33 and 5.86 |
| **27** | BGB 175 | 180 °C isothermal | 5.37 and 5.65 (4.21 and 4.42)* |
| **28** | BGB 175 | 180 °C isothermal | 4.29 and 4.73 |
| **29** | BGB 175 | 180 °C isothermal | 10.54 and 11.32 |

*****The determination of the *ee* of **27** using artUPO$_{bact}$ was conducted with a new BGB175 column, with adjusted retention times for enantiomers using the same conditions, as indicated.

## 10. Chiral GC Traces

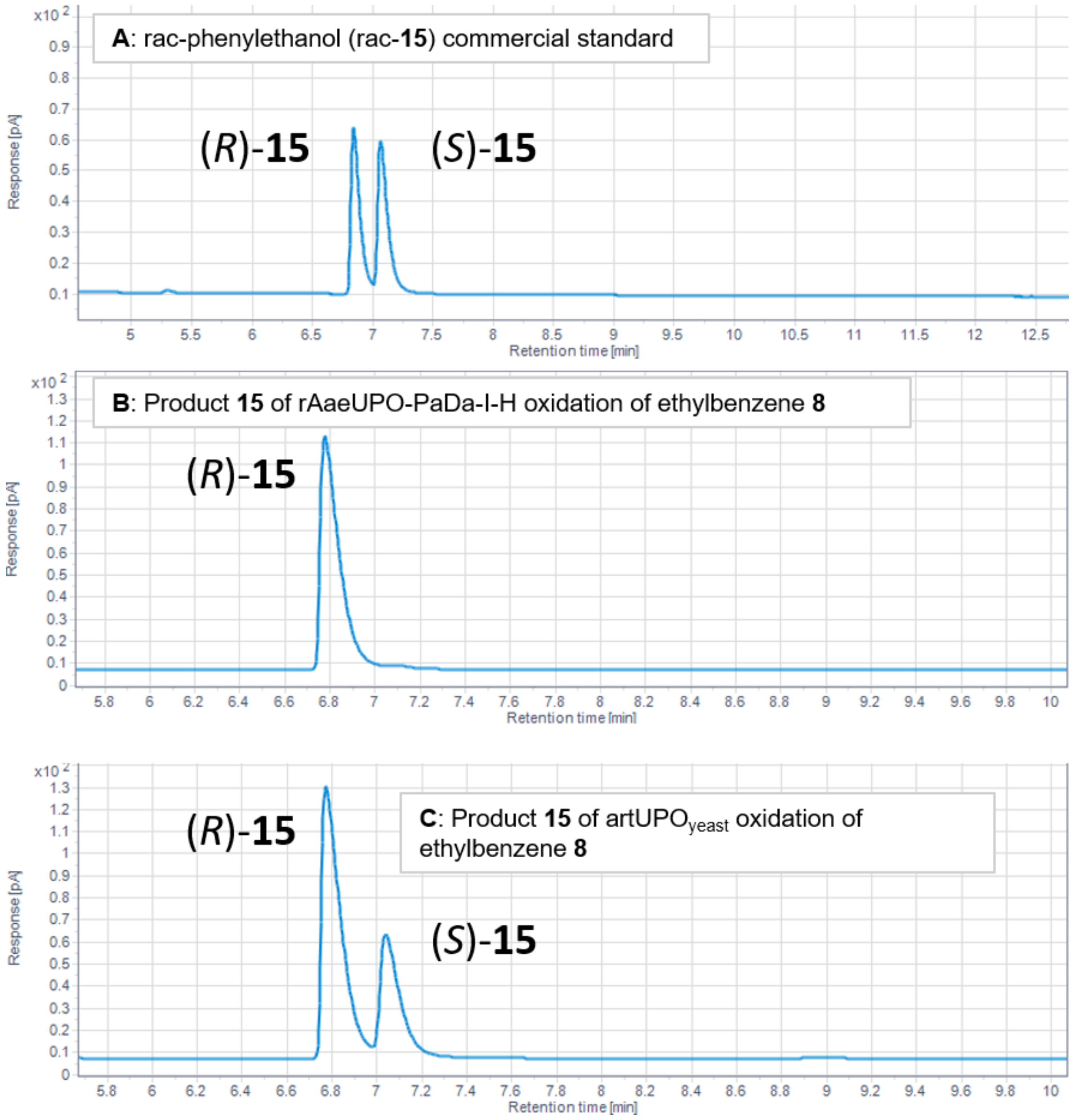


**Figure S11.** Chiral separation of enantiomers of **15**.

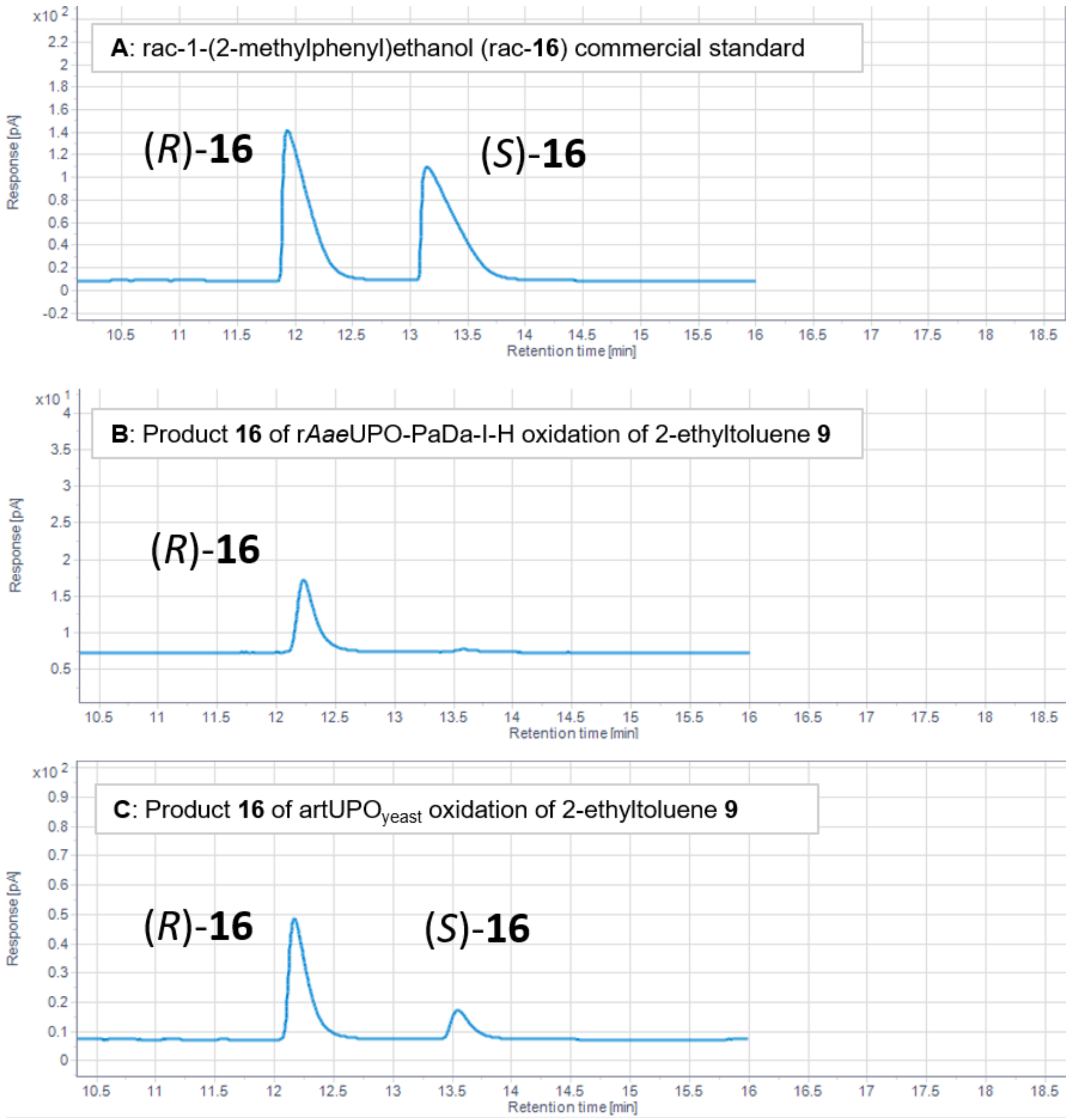


**Figure S12.** Chiral separation of enantiomers of **16**.

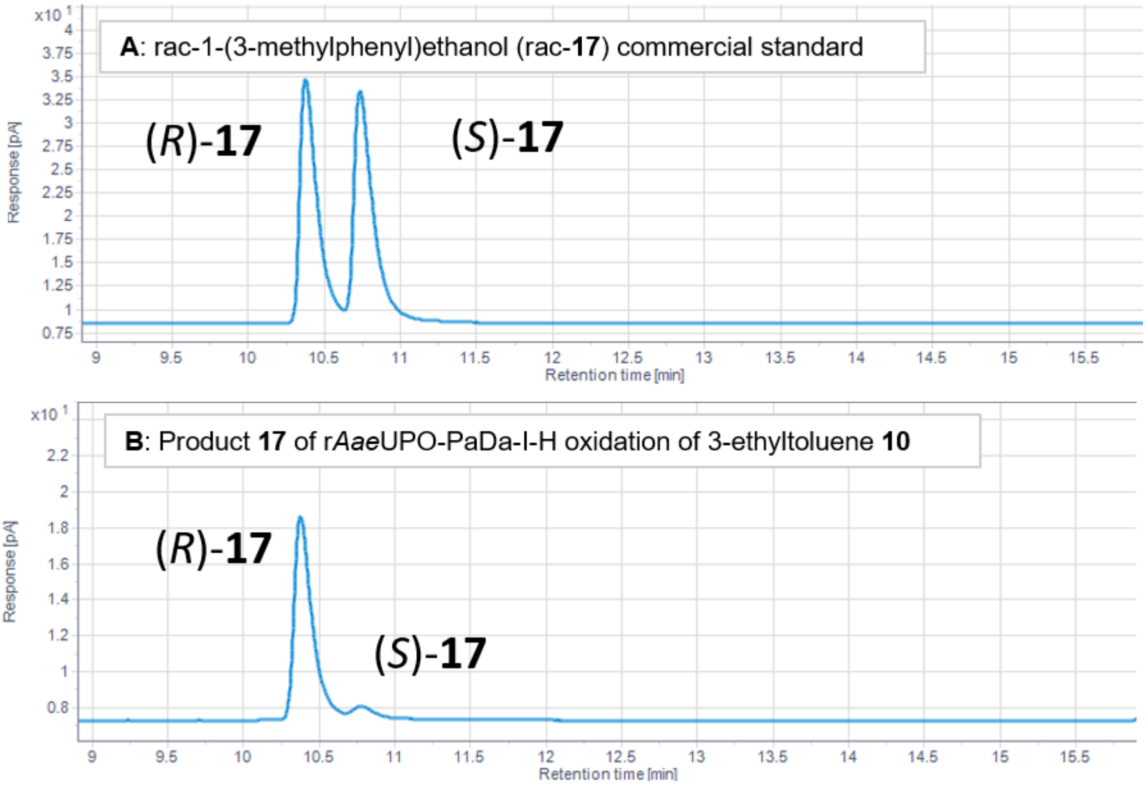


**Figure S13.** Chiral separation of enantiomers of **17**.

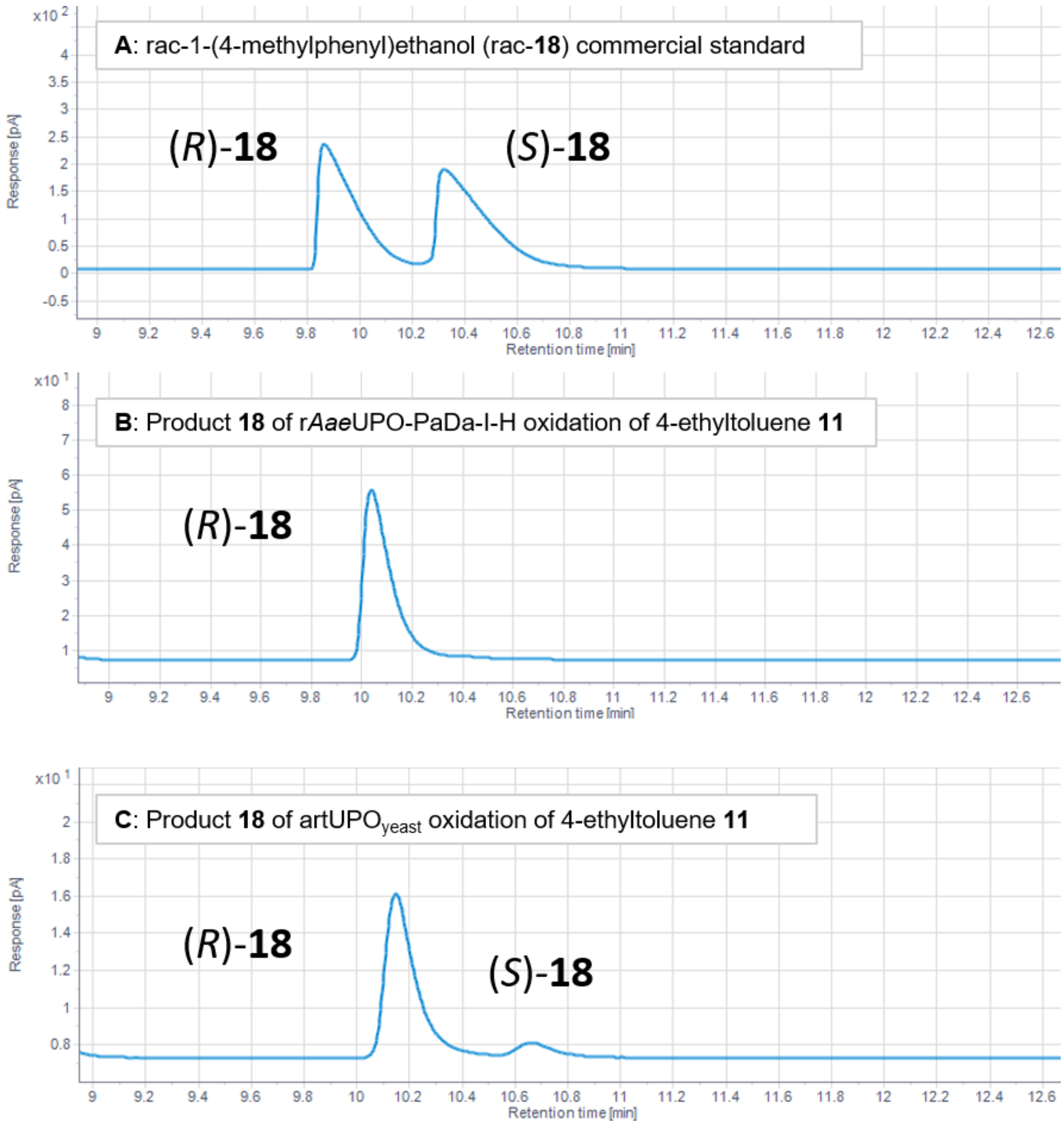


**Figure S14.** Chiral separation of enantiomers of **18**.

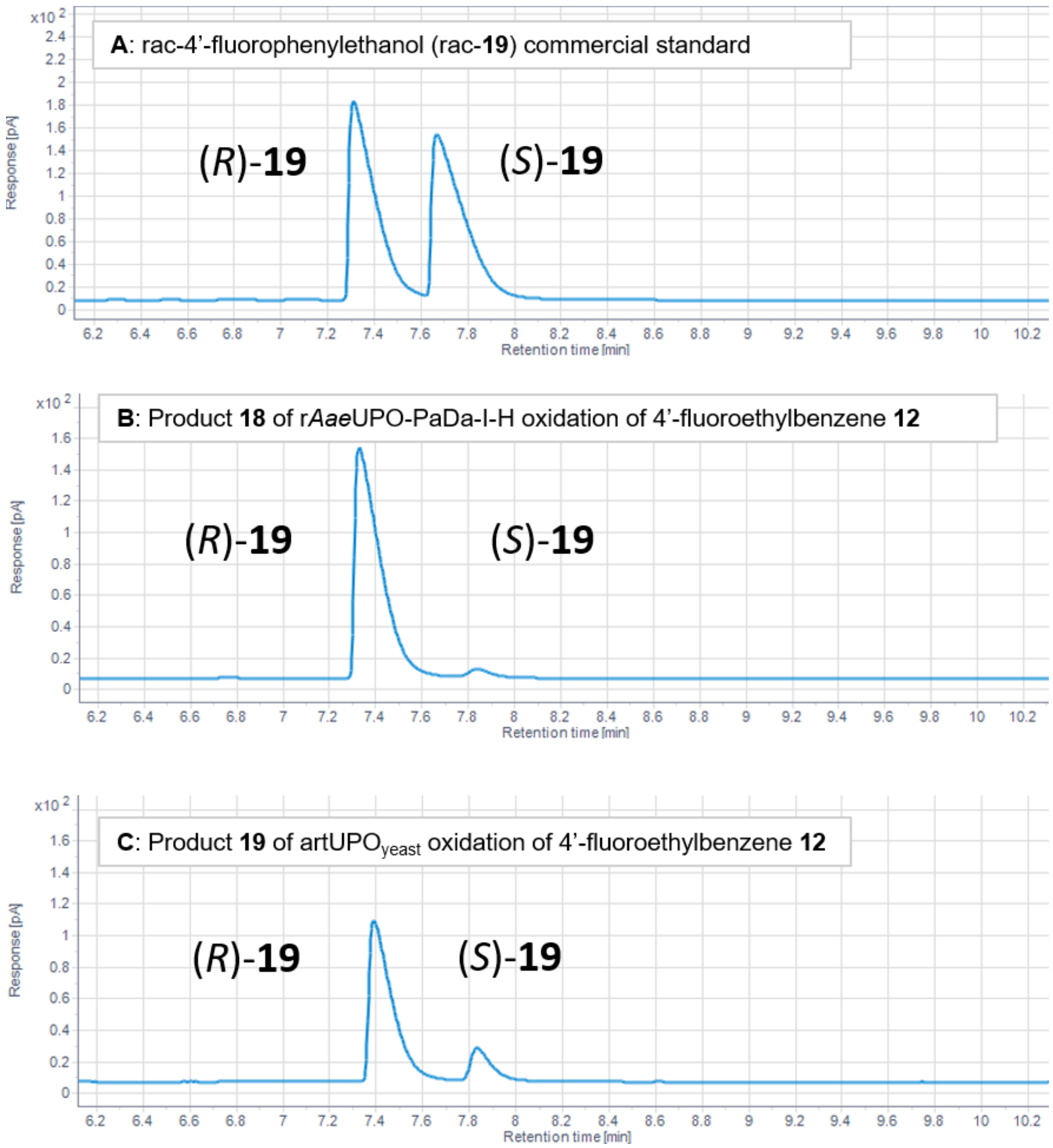


**Figure S15.** Chiral separation of enantiomers of **19**.

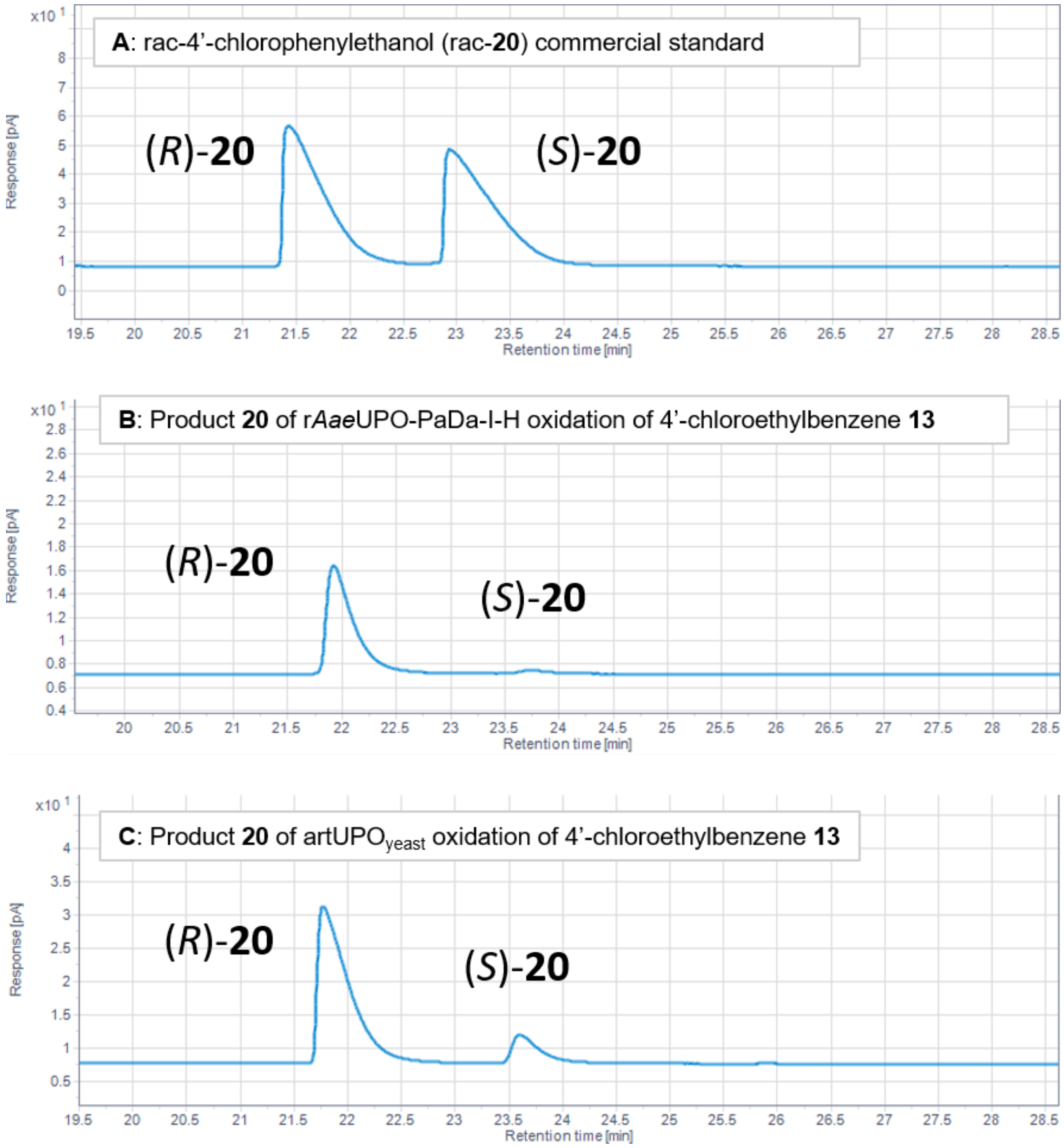


**Figure S16.** Chiral separation of enantiomers of **20**.

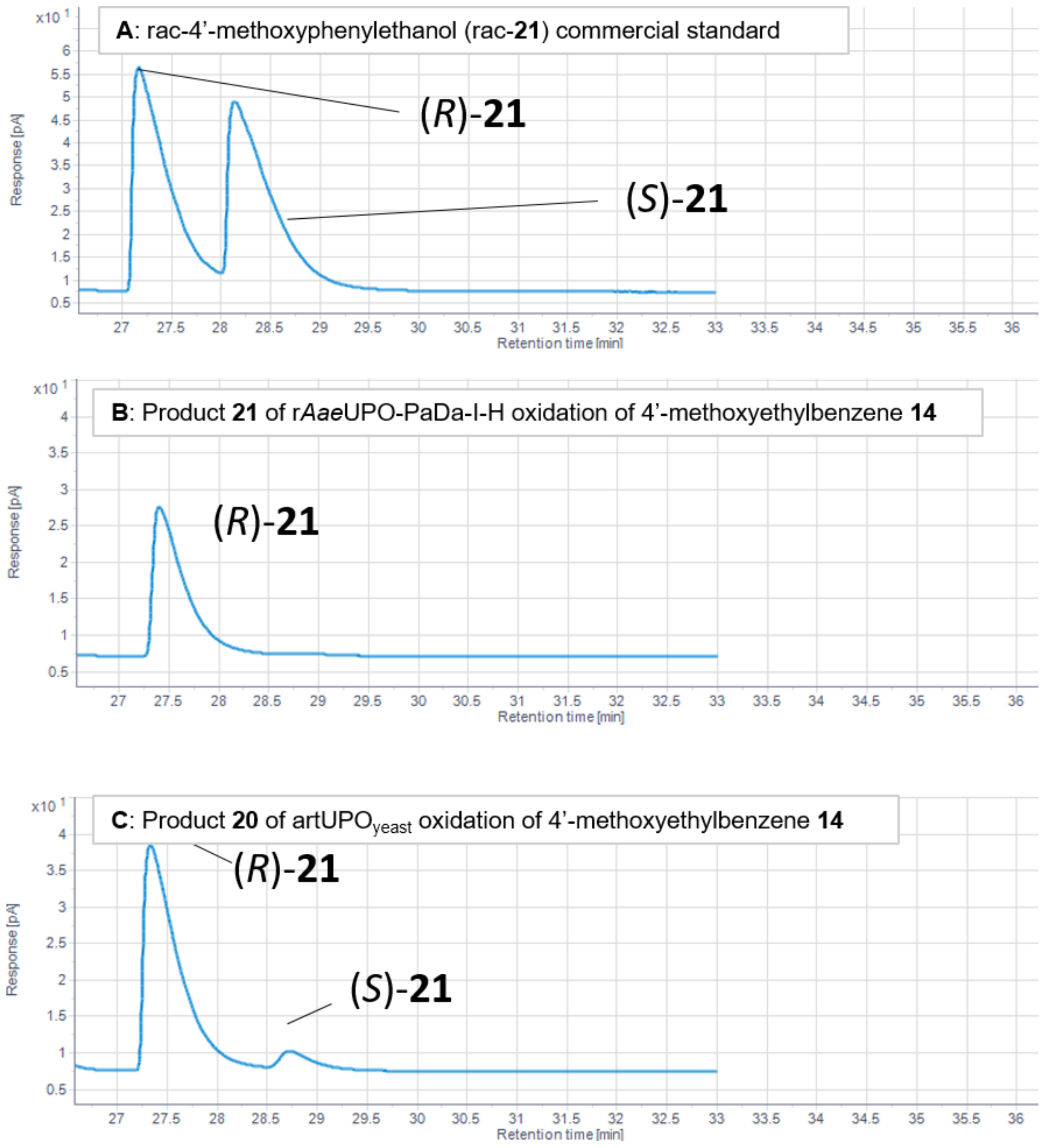


**Figure S17.** Chiral separation of enantiomers of **21**.

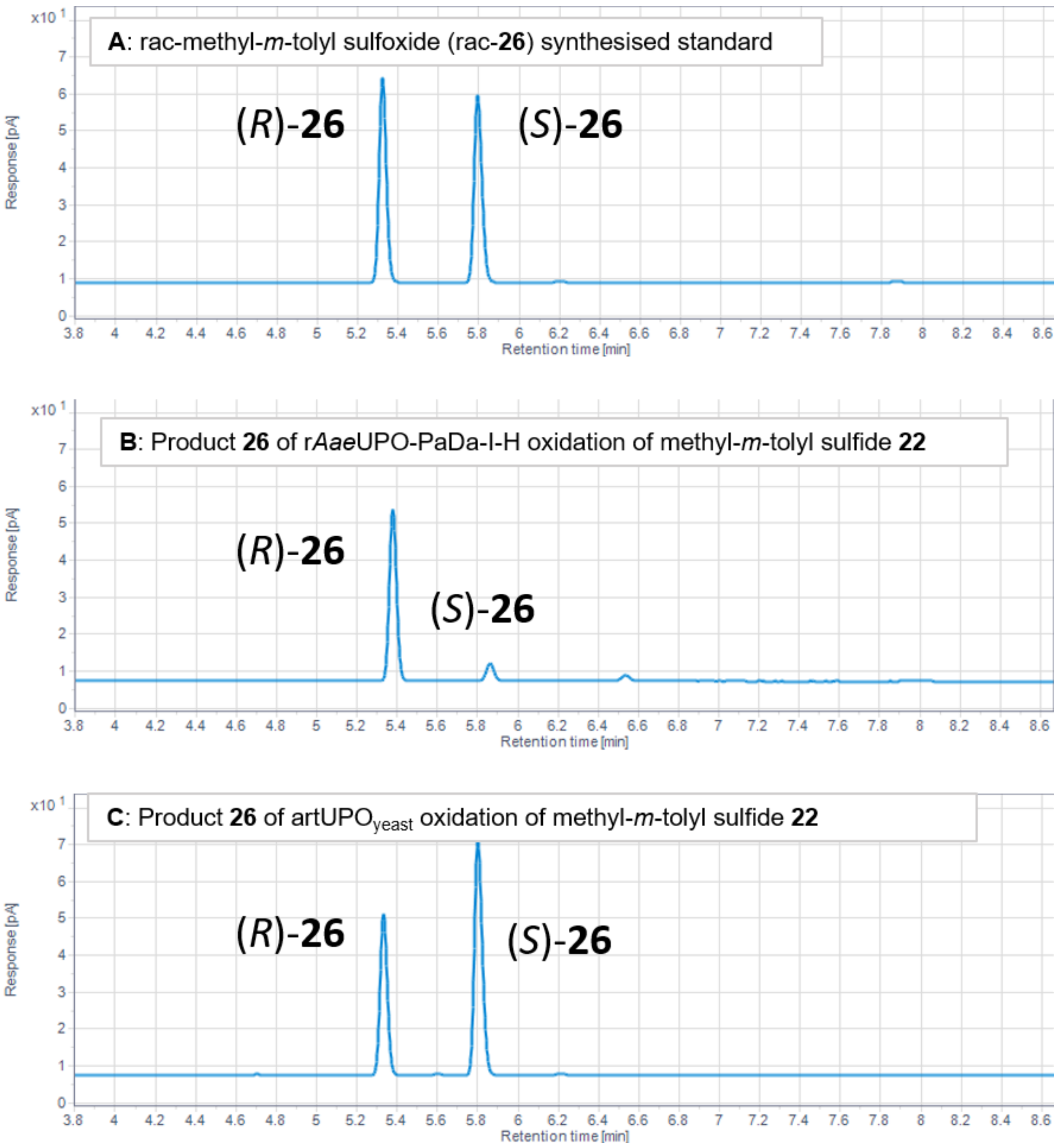


**Figure S18.** Chiral separation of enantiomers of **26**.

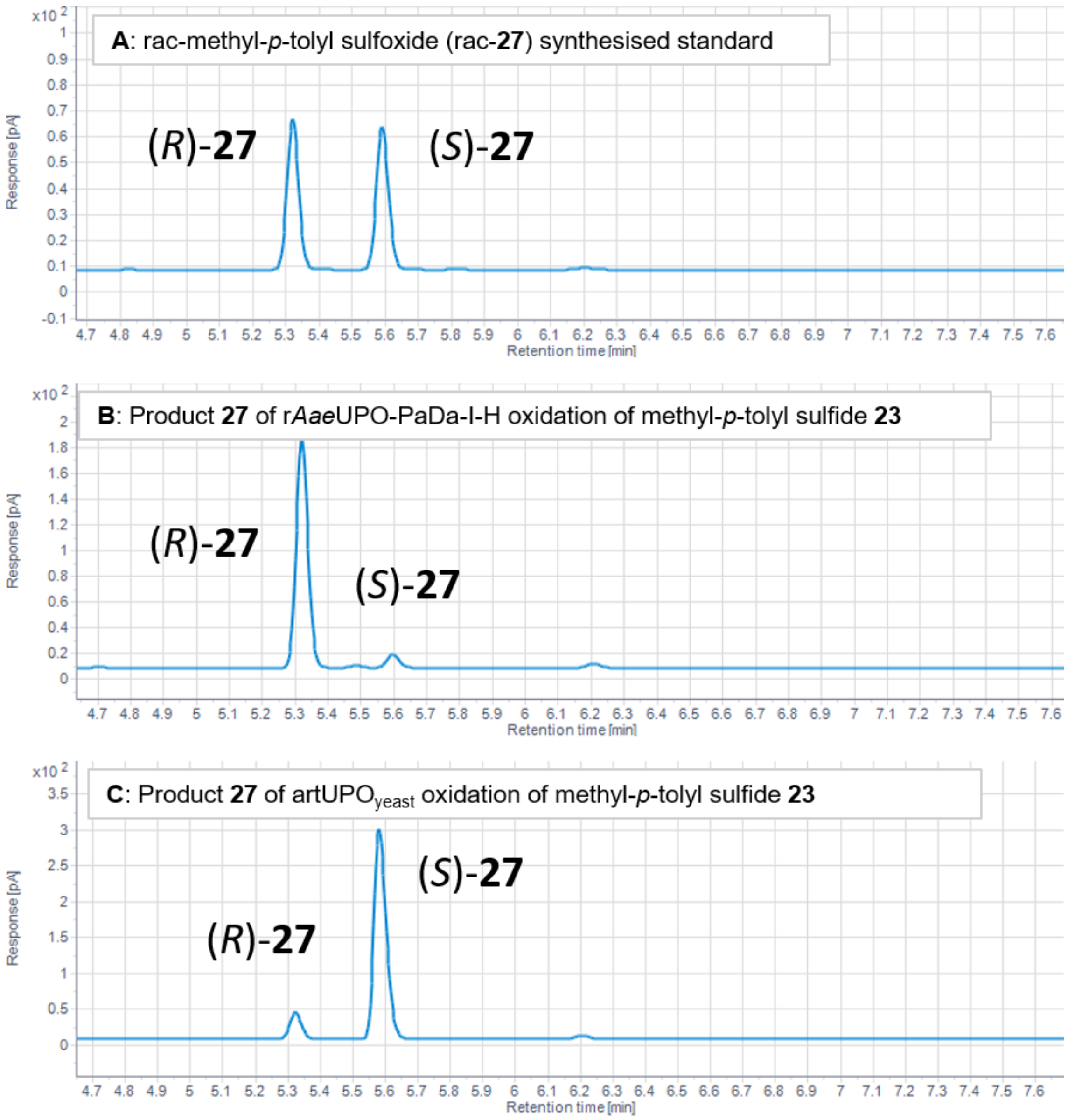


**Figure S19.** Chiral separation of enantiomers of **27**.

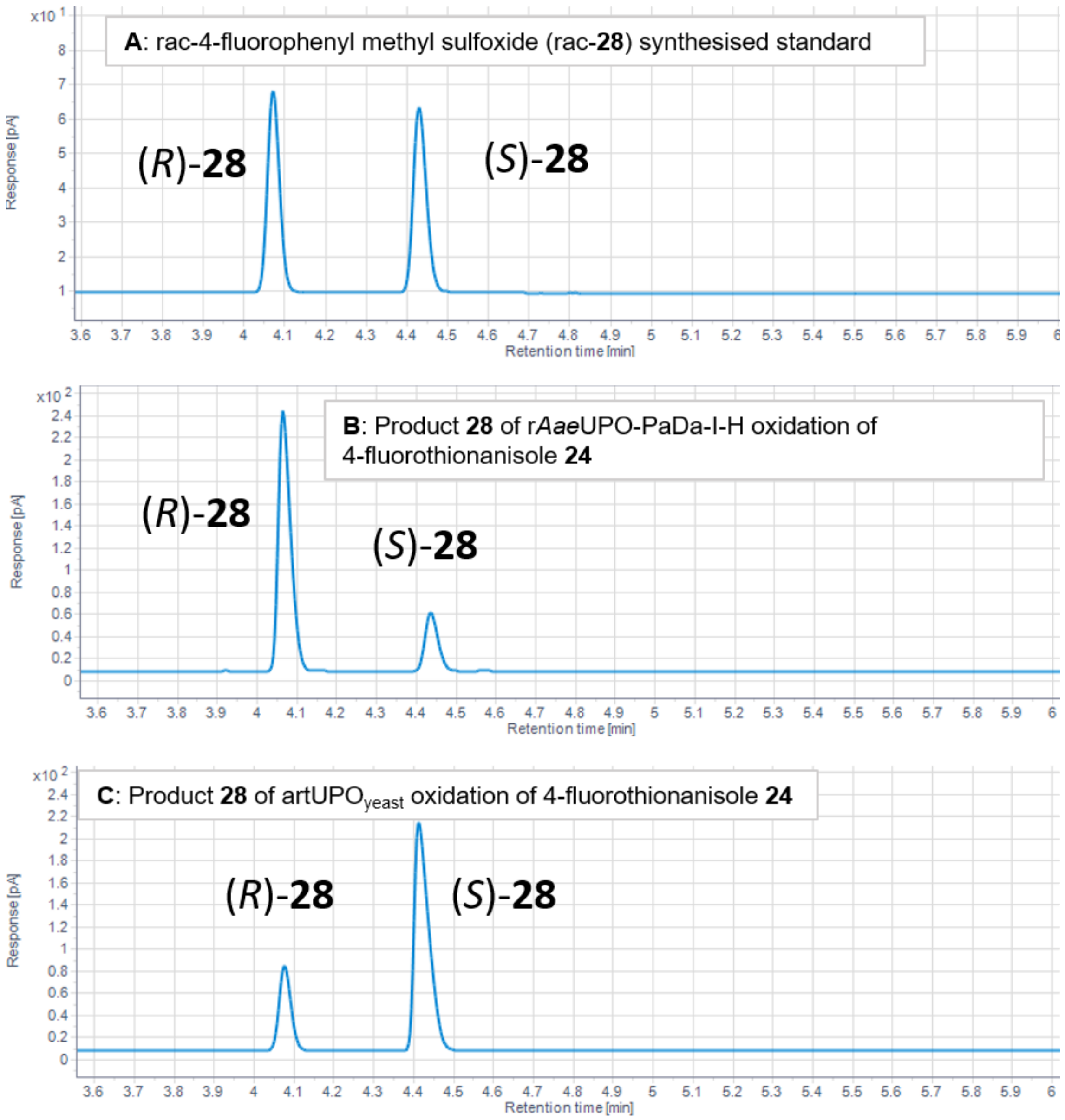


**Figure S20.** Chiral separation of enantiomers of **28**.

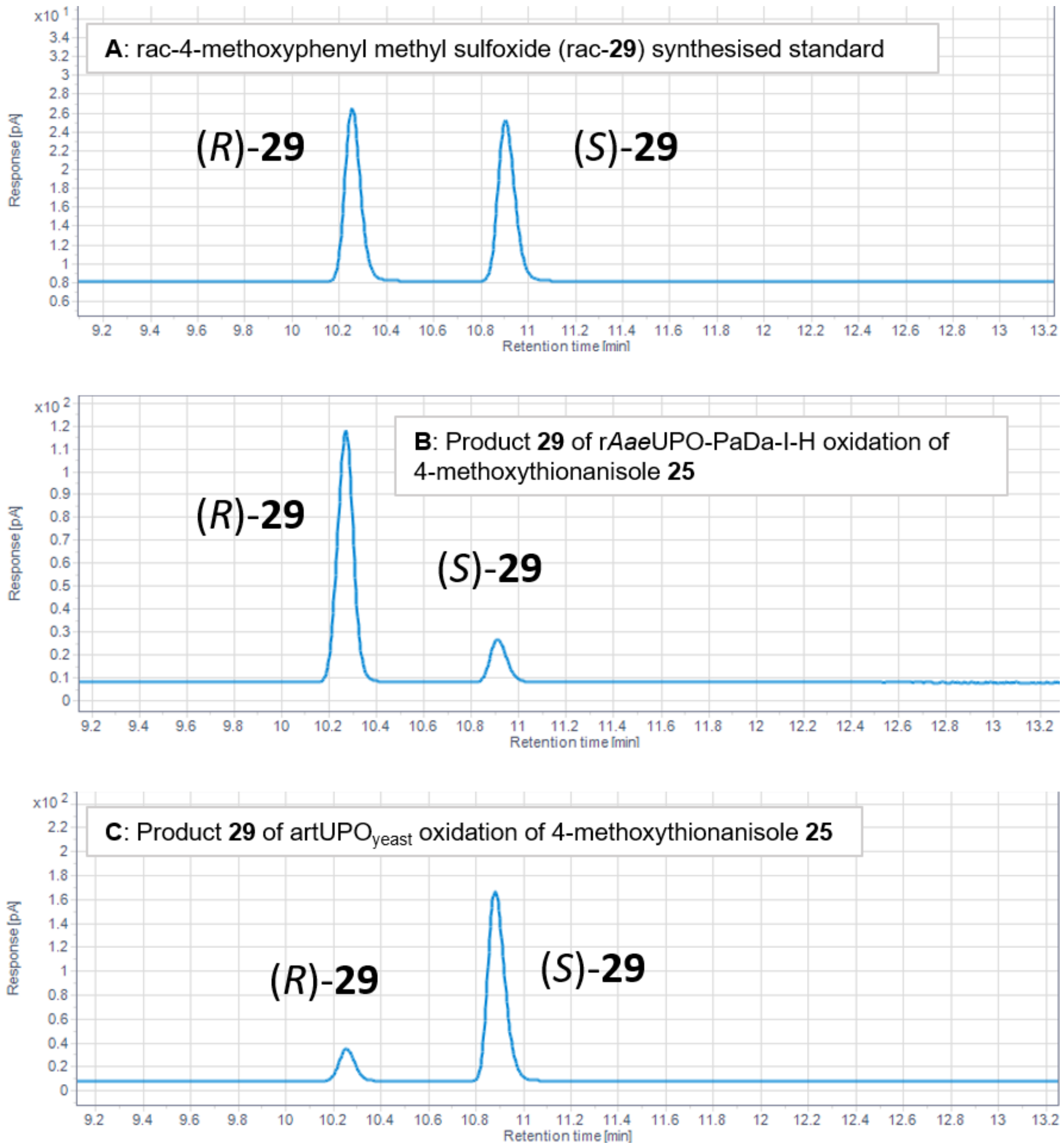


**Figure S21.** Chiral separation of enantiomers of **29**.

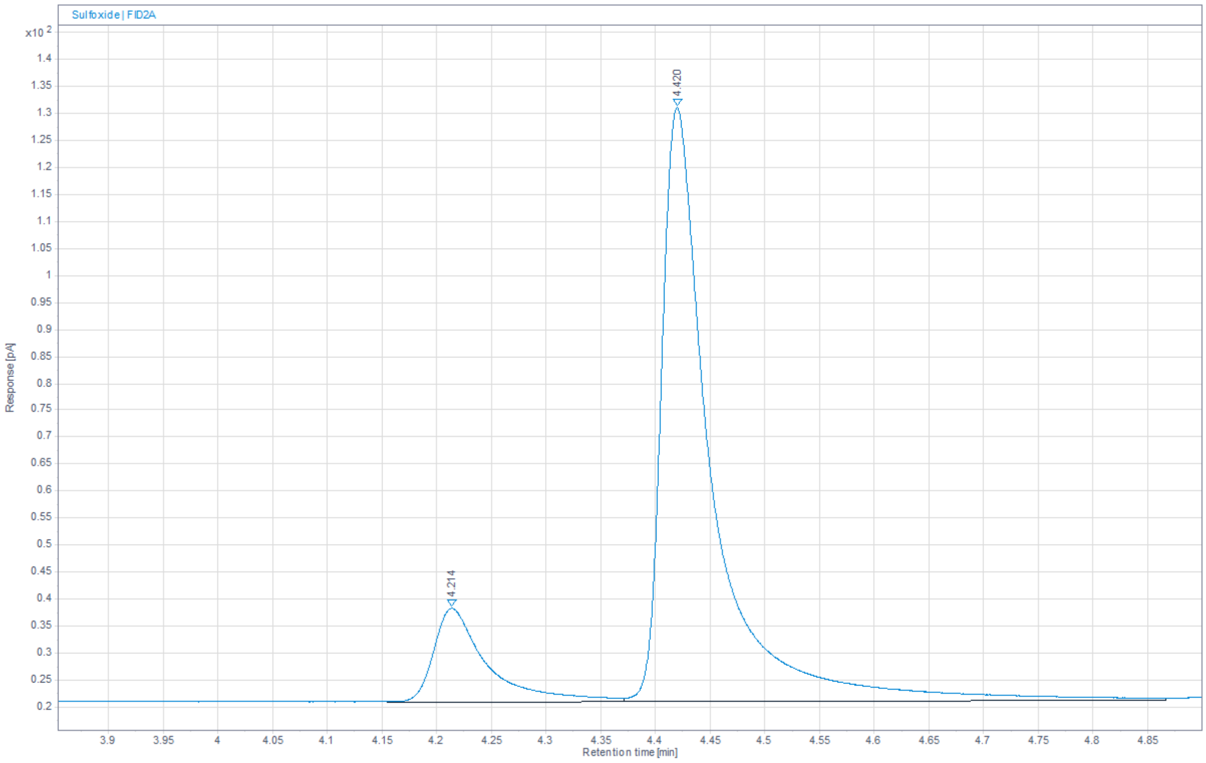


**Figure S22**. Chiral separation of enantiomers of **27** produced by artUPO$_{bact.}$
